# Seasonal and spatial variations of arsenic and its species in particulate matter in an urban environment of Brno, Czech Republic

**DOI:** 10.1007/s11356-024-34645-4

**Published:** 2024-09-03

**Authors:** Romana Michalicová, Jitka Hegrová, Josef Svoboda, Roman Ličbinský

**Affiliations:** Transport Research Centre, Líšeňská 33a, Brno, 636 00 Czech Republic

**Keywords:** Particulate matter, Arsenic speciation, Methylarsenic species, PM size distribution, ELPI +

## Abstract

The present paper deals with an analysis of total arsenic concentration using ICP-MS/MS and an analysis of concentration of several arsenic species, arsenite (As^III^), arsenate (As^V^), monomethylarsonate (MMA), dimethylarsenite (DMA), and trimethylarsine oxide (TMAO), using HPLC-ICP-MS/MS in the PM_10_ fraction of airborne urban aerosol. The samples were collected during two campaigns, in the autumn of 2022 and in the winter of 2023, at three locations within the central European city of Brno, with the aim to evaluate the seasonal and spatial variations in the PM_10_ composition. The results confirmed only the seasonal variability in the content of the methylated arsenic species in PM_10_ influenced by biomethylation processes. To gain better understanding of the possible arsenic origin, a supplementary analysis of the total arsenic concentrations was performed in samples of different size fractions of particulate matter collected using ELPI + . Local emissions, including industrial activities and heating during the winter season, were suggested as the most likely predominant source contributing to the total As content in PM_10_.

**Graphical abstract:**

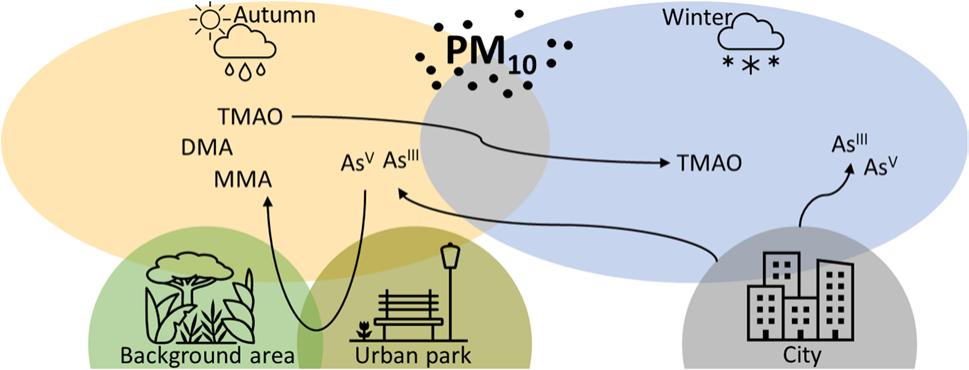

**Supplementary Information:**

The online version contains supplementary material available at 10.1007/s11356-024-34645-4.

## Introduction

Particulate matter (PM) is one of the basic indicators of ambient air pollution. It originates both from natural and anthropogenic sources. During the mid-nineteenth-century industrial revolution, anthropogenic PM became notably prevalent, especially in urban areas and in proximity to regional industrial sources (Fowler et al. 2020). The inhalable fraction consists of particles with an aerodynamic diameter of up to 10 µm (PM_10_). Because of their impact on human health (Goossens et al. 2021), the WHO global air quality guidelines (WHO 2021) set a recommended 24-h limit of 45 µg m^−3^ and annual limit of 15 µg m^−3^. The European and Czech legislative reflect these recommendations by implementing the 24-h emission limit of 50 µg m^−3^ with a maximum of 35 overruns per year and the annual limit of 40 µg m^−3^ in DIRECTIVE 2008/50/EC and Zákon č. 201/2012 Sb (n.d.), respectively. Thanks to the legislative and technical advances, PM emissions have been significantly reduced over the last decades.

PM is a complex mixture of airborne particles and liquid droplets of different sizes and chemical composition which depend on various factors, including the type of the emission source, the spatial and temporal distance from the source, interactions between local and far-range sources, and meteorological conditions. Therefore, knowing the particle size distribution can indicate the emission sources (Costabile et al. 2009; Watson et al. 2010). Also, the analysis of metal content in different size fractions of PM can be very helpful in identifying the emission source (Fomba et al. 2018; Matthews et al. 2022). For example, Costabile et al. (2009) conducted an extensive study of spatio-temporal variability and principal components of the particle number size distribution in an urban atmosphere of Leipzig in Germany. Thanks to their wide database and correlation with the literature they were able to develop a paradigm describing the evolution of the urban sub-micron aerosol. The studies of Fomba et al. (2018) and Matthews et al. (2022) analysing the PM size distribution focused on the metal content in individual size fractions with relation to sources in traffic. By analysing the known chemical tracer species, they were able to attribute PM to specific sources like road dust resuspension, combustion processes, and tire and brake wear.

Arsenic (As) is an element typically known for toxicity of its various species. In the atmosphere, this element is present either in the form of its volatile trivalent species or in liquid or solid state as a part of PM, where it is present in five basic forms. In its inorganic form, it is present as arsenite (As^III^) or arsenate (As^V^), while the organic forms include methylated species of pentavalent As, i.e., monomethylarsonate (MMA), dimethylarsinate (DMA), and trimethylarsine oxide (TMAO) (Jakob et al. 2010; Mukai et al. 1986). Of the As species listed above, the organic ones are generally considered less toxic. On the contrary, the inorganic species As^III^ is considered the most toxic of all the species (Huang et al. 2014; Sánchez-Rodas et al. 2015; Styblo et al. 2000). Hence, it is important to tell these various species apart. Many older studies focused only on the speciation of the inorganic As species (Rabano et al. 1989) or their ratio (González-Castanedo et al. 2015). However, some other studies focused also on the organic species (Lin et al. 2022; Mukai et al. 1986; Tanda et al. 2020; Tziaras et al. 2015; Yang et al. 2021). The formation of methylated species of As is called biomethylation or biovolatilisation due to the fact that various microorganisms, including algae, fungi, yeast and plants, can be their natural producers (Bentley and Chasteen 2002). Under favourable atmospheric conditions (at 20 °C in the dark), the volatile methylated arsines are relatively stable, with their half-life ranging from 19 weeks (arsine) to 2 days (trimethylarsine), but during daytime their stability drops by three orders of magnitude, probably due to photo-oxidative reactions with OH radicals. With an increasing number of methyl groups, the stability of the volatile As species decreases (Jakob et al. 2010). The arsine then oxidises to As^III^ or As^V^ and the trivalent methylated species oxidise to their pentavalent water-soluble analogues MMA, DMA, and TMAO (Haas and Feldmann 2000; Parris and Brinckman 1976).

In this paper, we focus on analysing the content of As and its species in PM_10_ in the environment of the central European city of Brno. First, with respect to traffic density, we hypothesise that the contamination by As will be stronger in the city centre than in the background area at the outskirts of the city due to the influence of traffic and industry. We also expect higher PM and As concentrations during the winter season due to emissions from household heating. Given that the organic As species are often discussed in the literature in the context of their biomethylation origin, we expect a higher proportion of these As species at locations rich in their bio-producers. Therefore, the specific environment of an urban park was chosen as a sampling area to test this hypothesis. Regarding the organic As species, we posit a more pronounced impact of seasonal trends at these locations; conversely, we anticipate a stronger impact of the inorganic As species within the city—closer to the anthropogenic pollution sources. For a deeper understanding of the PM origin, we decided to analyse the As content in different size fractions of PM as well. We have also decided to test a new As speciation HPLC method enabling a simultaneous separation of all five As species in one chromatographic analysis.

## Materials and methods

### Sampling locations

**Fig. 1:**
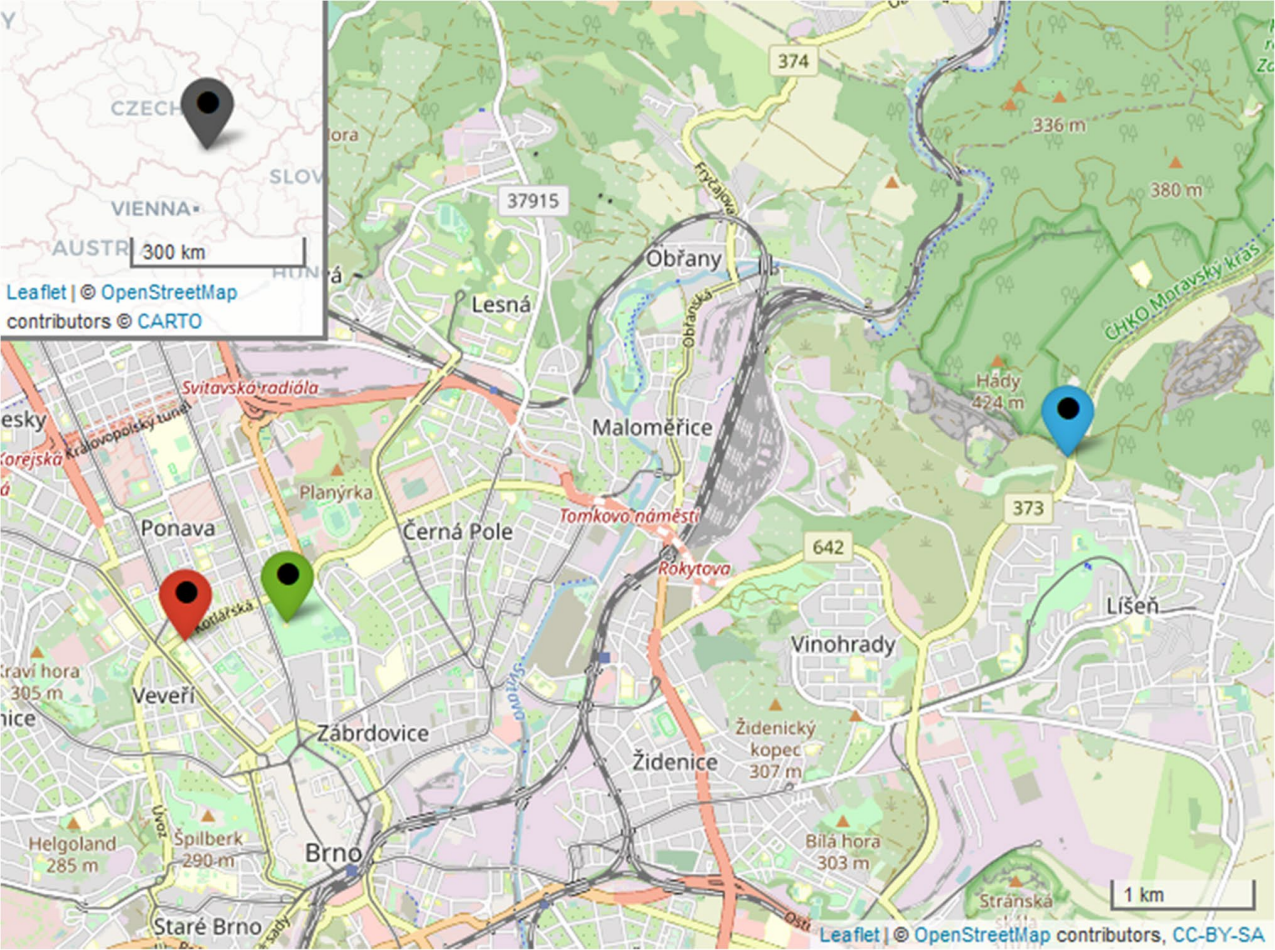
Sampling locations indicated on the map of the city of Brno. Locations: intersection KOT, red; urban park LUZ, green; background area KLA, blue (Cheng et al. 2022; OpenStreetMap n.d.)

Sampling of PM was performed at three locations in Brno (Fig. 1), which is the second largest city in the Czech Republic. Brno is home to 381 thousand residents, 66 thousand foreigners, and 70 thousand students (Intenzita dopravy n.d.); this diverse population lives within an area spanning 230 km^2^.

The first location (labelled “KOT”) was near the city centre, at the intersection of streets Kotlářská and Kounicova (49.2055019 N, 16.5972094 E, the altitude 242.6 m above sea level). Around 26 thousand vehicles pass through this intersection daily (Intenzita dopravy n.d.; ŘSD ČR n.d.). This location was chosen as one of the typical intersections in Brno with heavy traffic load.

The Lužánky Park (labelled “LUZ”) was chosen as a representative of an urban area unencumbered by heavy traffic. The park spans 22.3 hectares and is located near the city centre. Around 70 thousand visitors come to the park every year. The sampling location was situated approximately in the middle of the park, on the terrace behind the recreational centre building (49.2065895 N, 16.6069470 E, the altitude 212.8 m above sea level), around 120 m from the nearest adjacent road. The distance between the first two sampling locations was around 717 m.

The last location (labelled “KLA”), near the outskirts of the city, was chosen as the background area. The sampling was performed on the terrace of Velká Klajdovka Hotel (49.2167726 N, 16.6805594 E, an altitude 376.6 m above sea level). The distance from the other two locations was approximately 5.85 km.

### Sampling campaigns and meteorological conditions

Sampling was performed in two 14-day-long campaigns, one in the autumn of 2022 and the other in the winter of 2023.

The autumn campaign was conducted between October 6 and October 19, 2022. The average temperature in the Czech Republic reached 10.7 °C that month, which is markedly above the long-term average. The total monthly precipitation amounted to only 23 mm, which represents only 47% of the typical level. Dispersion conditions were normal (Škáchová Hana et al. 2022).

The winter campaign samples were collected in the period from January 31 to February 14, 2023. The conditions throughout February 2023 in the Czech Republic were assessed as normal with regard to temperature (monthly average of 1.2 °C), precipitation (total monthly rainfall of 37 mm), and dispersion conditions (Škáchová Hana et al. 2023).

### Sampling methods

Sampling of PM_10_ was performed at all three selected locations using nitrocellulose filters with a diameter of 47 mm and a pore size of 1.2 µm (Merck Millipore Ltd., Cork, Ireland). Each filter was used for a period of 24 h. In the autumn 2022 campaign, sequential samplers SEQ 47/50 (Sven Leckel Ingenieurbuero GmbH, Germany) were used for PM_10_ sampling, while samplers MVS6 (Sven Leckel Ingenieurbuero GmbH, Germany) were used for the PM_10_ sampling during the winter 2023 campaign. The airflow rate was kept at 2.3 m^3^ h^−1^ (≈ 55.2 m^3^ per 24 h).

At the LUZ location, in addition to PM_10_, different PM size fractions (PM_x_) were sampled using ELPI + low-pressure impactor (Decati Ltd, Kangasala, Finland), which enables simultaneous measurement of fourteen size fractions in the size range from 0.006 to 10 µm (fourteen impactor stages 0.016, 0.030, 0.054, 0.094, 0.15, 0.25, 0.38, 0.60, 0.94, 1.6, 2.5, 3.6, 5.3, and 10 µm and one filter stage 0.006 µm). For PM_x_ sampling, polycarbonate collection foils (diameter 25 mm; Whatman® NucleporeTM, GE Healthcare, USA) were used, previously exposed for the entire 14-day period (with an airflow rate of 0.602 m^3^ h^−1^ ≈ 200 m^3^ of filtered air per foil).

### Filter processing and As extraction

The exposed filters were stored at constant laboratory temperature, in the dark in PetrislidesTM (Merck KGaA, Darmstadt, Germany) prior to the analysis.

After gravimetric determination of the content of PM_10_, each nitrocellulose filter was cut into three pieces and each part was weighed using analytical microbalances MX5 (Mettler-Toledo GmbH, Switzerland). Each part was used for a different purpose. Since the cutting was done manually using ceramic scissors, the weight of the individual parts may not be the same. To ensure the accuracy of the results, when relating the final total As concentration and the concentration of As species to the total volume of air filtered or to the total PM_10_ content, the cutting factors were calculated as the ratio of the weight of the whole filter against the weight of the specific part.

The first part of the filter was used for the determination of the total As content in the PM_10_ sample. For this purpose, it was digested using the microwave system SW-4 (Berghof, Germany) in a mixture of 5 ml HNO₃ subboiled (Distillacid, Berghof GmbH, Germany) and 1 ml of H_2_O_2_, 30%, ultrapure grade (ANALYTIKA®, Czechia). The ELPI + polycarbonate foils were digested in the same way with only one difference—they were processed as a whole, without cutting to smaller pieces. Details are available in Supplementary Information (SI), Table S1.

The second part of the nitrocellulose filter was extracted by ultrapure grade water (18.2 MΩ cm; Simplicity UV, Merck, Germany), since water is considered to be the simplest representative of human lung fluid for bioavailability testing (Cigánková et al. 2021), and the extract was used for the As speciation analysis. The filter, or rather the part thereof, was placed in a 15 ml transparent centrifuge tube (Wuxi NEST Biotechnology Co., Ltd, China) and immersed in 1 ml of ultrapure water (18.2 MΩ cm; Simplicity UV, Merck, Germany). After sealing, the tube was placed into an ultrasonic bath at 60 °C and sonicated for 1 h. To cool down the extract and facilitate the subsequent filtration, the sonicated sample was centrifuged using the Universal 320R centrifuge (Andreas Hettich GmbH & Co. KG, Germany) and afterwards filtered through a 0.2 µm Millex® PTFE syringe filter (Merck Millipore, Germany).

The last part of the nitrocellulose filter was stored for the speciation of chromium, which is not the subject of this publication.

### Determination of total PM_10_

The total concentration of PM_10_ in the air was determined by the gravimetrical method according to the ČSN EN 12341 (n.d.) standard. Each nitrocellulose filter was weighted before and after exposure using MX5 microbalances (Mettler-Toledo GmbH, Switzerland). The weight difference corresponding to the amount of PM_10_ was related to the total volume of filtered air (typically approximately 55.2 m^3^ per 24 h and per filter).

### Total As analysis

The total As concentration was determined in the microwave-digested samples and in the water extracts. The analysis was performed using an inductively coupled plasma mass spectrometer with triple quadrupole (ICP-MS/MS) Agilent 8800 (Agilent Technologies, Germany).

For As observation, the MS/MS scan in O_2_ reaction mode was chosen, where AsO^+^ is formed in an octopole reaction cell (ORC) and detected at mass 75 → 91 with an integration time of 0.3 s/mass. The O_2_ gas flow rate was set at 0.29 ml/min. Because the estimated concentrations in the samples were very low, especially in the ELPI + samples, the plasma was set to a low matrix mode.

An internal standard solution was used for the correction of any ICP-MS/MS fluctuations during the analysis. It was prepared by diluting the Internal Standard Mix (Agilent Technologies, Germany) to a concentration of 10 µg l^−1^ in 2% HNO₃ subboiled (Distillacid, Berghof GmbH, Germany). Yttrium at the mass shift 89 → 105 (integration time/mass, 0.1 s) was selected as the internal standard element.

Calibration was performed in the range of 0.01–100 µg l^−1^. Calibration solutions were prepared by diluting a single-element certified As reference material with a concentration of 1000 ± 2 mg l^−1^ (ANALYTIKA®, Czechia) in 2% HNO₃ subboiled (Distillacid, Berghof GmbH, Germany).

Each sample was automatically measured three times by the instrument and the final As concentration was expressed as a mean with standard deviation. The results were then related to the amount of PM_10_ captured on the filter or to the volume of air filtered and expressed in units of ng As mg^−1^ or pg As m^−3^, respectively.

The measurement trueness was verified by simultaneous analysis of certified reference materials: 1640a (National Institute of Standards & Technology) with a certified value of 8.075 ± 0.070 µg As l^−1^ and TM 35.2—trace elements in water (Labmix24 GmbH, Germany) with a target value of 7.02 ± 0.55 µg As l^−1^. Both were diluted 10 times with ultrapure water prior to the analysis (SI, Table S2 and Fig. S1).

The limits of detection (LoD) and quantification (LoQ) were calculated as the mean of 40 replicates of the measured blank sample (2% HNO_3_ subboiled) concentration increased by triple the standard deviation for LoD and tenfold the standard deviation for LoQ. The blanks were continuously added to the measuring sequence (SI, Table S3).

### As speciation analysis

The distribution of As in the form of its individual species (As^III^, As^V^, MMA, DMA, and TMAO) was observed in the water-extracted PM_10_ samples using the HPLC-ICP-MS/MS method.

The separation was performed in the gradient of two ammonium bicarbonate mobile phases (MP), both with the same pH but different concentrations (10 mmol l^−1^ and 100 mmol l^−1^). To prepare the more concentrated MP, ammonium bicarbonate (BioUltra, ≥ 99.5% (T); Sigma-Aldrich, Germany) was dissolved in ultrapure water (18.2 MΩ cm; Simplicity UV, Merck, Germany) and the pH was adjusted to 9.00 using 25% ammonium solution (Merck, Germany). The second MP was prepared from the concentrated one by dilution and subsequent pH control with readjustment, if needed, using the ammonium solution in case of deviation from the required value of 9.00. To the gradient was added 3% of methanol (HPLC grade, ultrapure G, Chromservis s. r. o., Czech Republic). The gradient used is given in SI, Table S4.

The As was detected using ICP-MS/MS operated under conditions analogous to the previous chapter, with the only difference that the low matrix mode was not used.

The HPLC instrument used was Agilent 1260 Infinity II (Agilent Technologies, Germany) with Hamilton PRP-X100 anion-exchange chromatographic column (250 × 2.1 mm, pore size 5 µm, stainless steel; Sulpeco, USA) and PRP-X100 guard column (10 µm, PEEK; Sulpeco, USA). The column holder temperature was maintained at 40 °C throughout the analysis.

Calibration was performed in the range of 0 to 50 µg As l^−1^. Mixed calibration standards were prepared using the following standard solutions: As^III^ Standard for ICP (1002 ± 4 mg l^−1^; Sigma-Aldrich, USA), As^V^ Standard for ICP (995 ± 3 mg l-1; Sigma-Aldrich, USA), MMA Standard (disodium methyl arsonate hexahydrate; Chem Service, USA), DMA (dimethylarsinic acid; Chem Service, USA), and TMAO Standard (trimethylarsine oxide; Toronto Resesarch Chemicals, Canada).

### Statistical analysis

The data evaluation and statistical analysis were performed with the OriginPro 2024 (64-bit) SR1 software, version 10.1.0.178. The influence of individual factors, like the sampling location or the season, was evaluated via one-way analysis of variance, ANOVA, at a significance level of 0.05. The results are available in SI.

## Results and discussion

### Total As content in PM

**Fig. 2:**
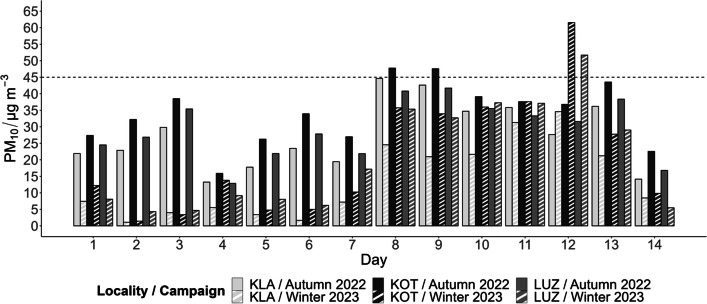
Total PM_10_ concentrations measured during the two sampling campaigns at the three selected locations in units of µg PM_10_ per m^3^ of filtered air. The dashed line represents the limit for 24-h mean recommended by the WHO (WHO 2021). Source data can be found in the SI, Table S5

The average of all the observed PM_10_ concentrations in the air was 24.3 ± 14.1 µg m^−3^ which is consistent with typical values for European cities (Querol et al. 2004). The limit for the 24-h mean recommended by the WHO is 45 µg m^−3^ (WHO 2021) and this limit was exceeded only four times, on two consecutive days during the autumn campaign at the KOT location with heavy traffic load and once during the winter campaign, simultaneously at the two locations inside the city, KOT and LUZ (Fig. 2).

However, the autumn/winter proportion in the results was atypical. In the winter, PM content in the air usually increases due to household heating (Jiao et al. 2023), but our data show an opposite trend. The total autumn concentration was almost twice as high as the winter concentration. The seasonal difference was statistically confirmed at the 0.05 level of significance (SI, Table S6 and Fig. S2). This was caused by a decrease in the first week of the winter campaign influenced by occluding frontal systems crossing the Czech Republic from the northwest. Due to the cold and humid ocean current, concentrations were well below the emission limit (Škáchová Hana et al. 2023). This fact needs to be taken into consideration for further evaluation of the results.

**Fig. 3:**
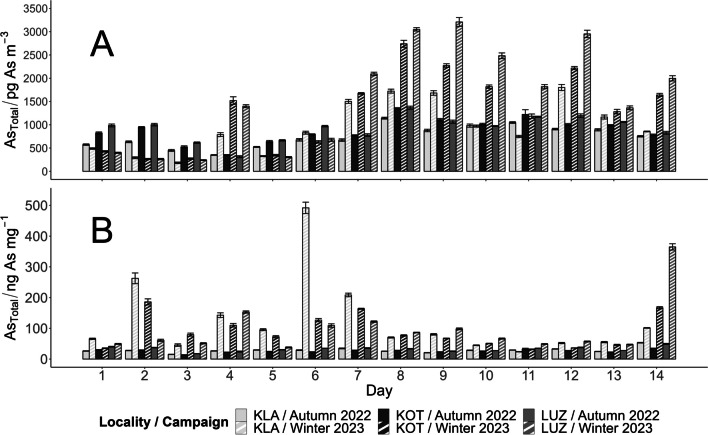
As_Total_ concentrations in mineralised samples collected during both sampling campaigns for the three selected sampling locations in units of pg As per m.^3^ of filtered air (**A**), ng As per mg of PM_10_ (**B**). Source data can be found in the SI, Tables S6 and S7. The error bars represent measurement standard deviations. Source data are available in SI, Table S7 and S8

The total As (As_Total_) concentrations analysed in the mineralised PM_10_ samples followed an analogous trend to PM_10_ (Fig. 3A). The lowest As_Total_ concentrations were observed in autumn (852 ± 263 pg As m^−3^). The results measured in winter samples were higher and also showed a bigger dispersion (1282 ± 867 pg As m^−3^) caused by the difference in the total PM_10_ concentrations between the first and the second week of the PM_10_ sampling period described above. The winter increase in As_Total_ content is clearly visible when related to the total PM_10_ concentration (Fig. 3B). The seasonal difference was statistically confirmed at the 0.05 level of significance (SI, Table S9 and Fig. S3). Hence, even though the total PM_10_ in autumn outweighed the winter concentrations, the As_Total_ content in PM_10_ was higher in winter, because the sources of PM changed with the season. Household heating during the period of low outside temperature is the most likely source (Jiao et al. 2023).

The KLA location in the outskirts of Brno showed a background character as expected, with the lowest concentrations of As_Total_ (851 ± 428 pg As m^−3^). However, a comparison of the remaining two locations in close proximity to the city centre revealed a rather surprising outcome. The highest As_Total_ contamination was observed at location LUZ in the middle of the park (1257 ± 847 pg As m^−3^). The As_Total_ contamination at the crossroad with heavy traffic load, KOT, was slightly lower (1093 ± 636 pg As m^−3^). Statistical comparison of sampling locations (SI, Table S10 and Fig. S4) showed no difference at the 0.05 significance level. This implies that traffic was not the main source of As pollution, which supports a similar conclusion made by Dousova et al. (2020), who monitored As content in a road dust and urban topsoil in three cities in the Czech Republic, and Karagulian et al. (2015), who attributed only 8% of PM_10_ to originate from traffic in central and eastern Europe. According to these authors, industry (18%), domestic fuel burning (45%), and other unspecified anthropogenic sources (26%) were stronger sources of PM_10_ emissions. A map showing possible sources of PM in Brno is included in the SI, Fig. S5 and Table S11. The closest source to the sampling locations is the heating plant, approximately 1 km north, producing 2.192 t of PM emissions per year (CHMI 2022 n.d.). Under certain meteorological conditions, airborne PM emissions can spread to the south and increase the PM concentrations at the city park, while the other location (KOT) is protected behind a barrier of city housing blocks built between the site and the heating plant (Hao et al. 2019).

**Fig. 4:**
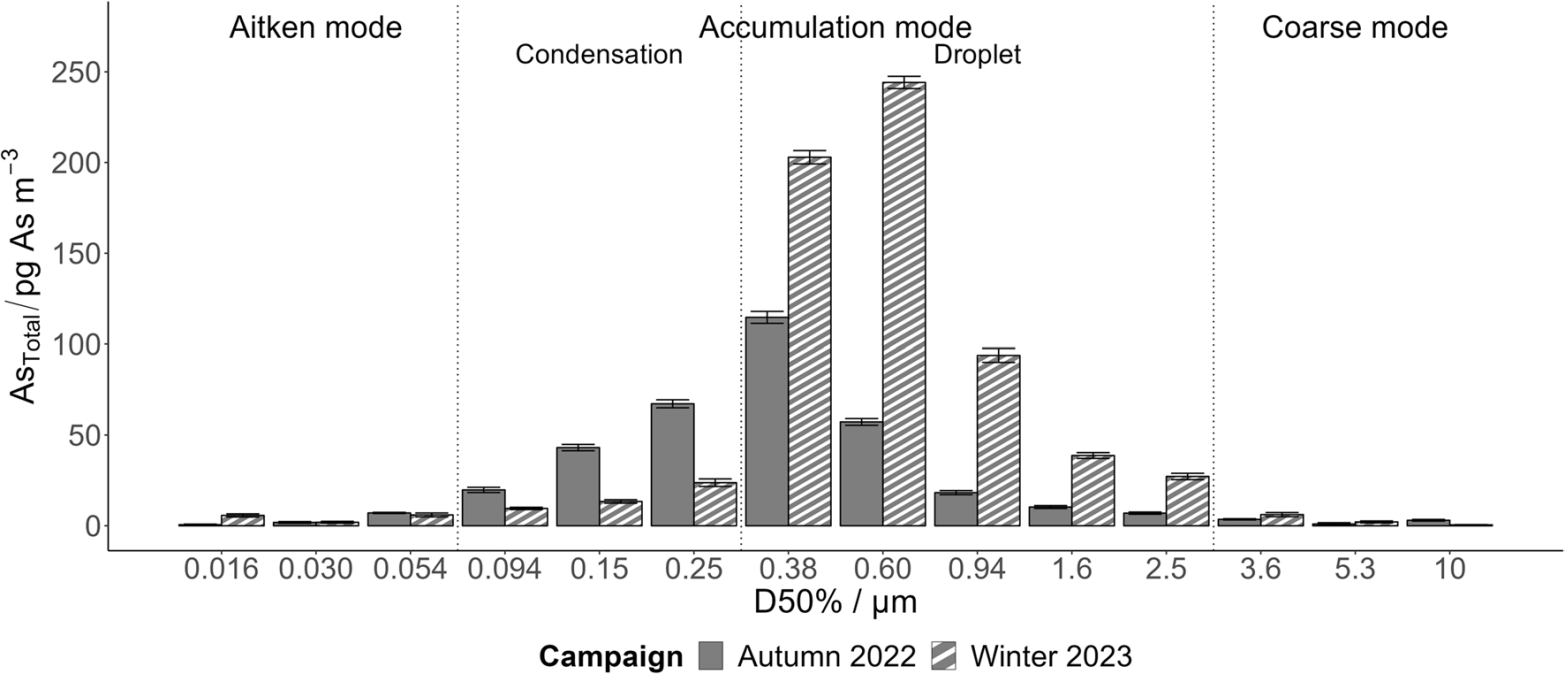
The As_Total_ distribution across the 14 size fractions of PM_x_ sampled using the low-pressure impactor ELPI + . Comparison of samples collected at the LUZ location during both sampling campaigns. Source data are available in the SI, Table S12

The analysis of As_Total_ content in the 14 above-mentioned size fractions of PM_x_ was performed to provide supplementary information about its possible sources. The greatest concentration of As_Total_ was observed in size fractions from 0.094 to 2.5 µm (Fig. 4), falling mostly into the accumulation mode (Watson et al. 2010). Similar findings were presented for example in (Savage et al. 2017).

During the autumn campaign, the maximum load of As_Total_ was present in the fraction with an aerodynamic diameter of 0.38 µm (≈ 32% of total As). Ninety percent of As_Total_ across all 14 sizes of PM_x_ was contained in fractions with aerodynamic diameters ranging from 0.094 to 0.94 µm.

In the winter campaign, the highest As_Total_ concentration was observed in the fraction with an aerodynamic diameter of 0.60 µm (≈ 36% of As_Total_). Ninety percent of the As_Total_ across all 14 PM_x_ fractions was present in particles with diameters ranging from 0.25 to 2.5 µm. The As_Total_ distribution across the 14 size fractions was shifted overall towards larger diameter particles (Fig. 4), slightly increasing the proportion in the droplet mode to the detriment of the condensation mode.

According to the study by Costabile et al. (2009), the traffic and the ageing of urban particles by condensation and coagulation processes are the likely sources of condensation mode particles. However, since we have already eliminated the traffic as the main source, it is more likely caused by the regional primary sources of urban particles (see SI, Fig. S5). The droplet mode particles are typically produced in clouds and fog under high-humidity meteorological conditions (Costabile et al. 2009; Noble and Hudson 2019), which are not unusual for the winter period in central Europe in recent decades.

### Arsenic species in PM_10_

In previously published studies of HPLC-ICP-MS As speciation in airborne aerosol were often used methods using Hamilton PRP-X100 anion-exchange column in combination with phosphate buffer mobile phase (Oliveira et al. 2005; Tanda et al. 2019, 2020). A disadvantage of this method is a coelution of As^III^ and TMAO peaks near the void volume of the column. A possible solution is to add a preparative step to the analysis and repeat the analysis after oxidation of As^III^ to As^V^, which extends the analysis time and creates room for the possible introduction of errors. Some studies even introduce the oxidation step prior to the analysis and determine the As^III^ and As^V^ species only as one common category of total inorganic As (Tanda et al. 2020). Other authors decided on the solution including a second separation method with a cation exchange column which enables additional separation of the As^III^ and TMAO peaks (Xie et al. 2021). Tziaras et al. (2015) came up with a unique method with tandem of anion and cation exchange chromatographic columns, enabling them the separation of all five As species during one chromatographic analysis.

Bicarbonate buffer has already been used as MP in combination with PRP-X100 column for HPLC separation of As species in different kinds of matrices, like macroalgae or urine samples (Reid et al. 2020). Xie et al. (2021) speciated this way As even in atmospheric PM. They used isocratic elution for the separation of species MMA, DMA, and As^V^, but for the separation of As^III^ and TMAO they used different chromatographic separation method with cation exchange column.

**Fig. 5:**
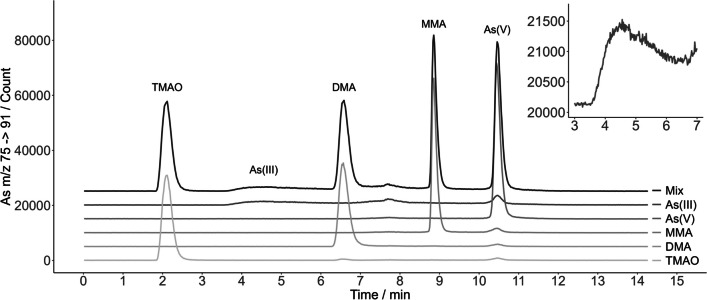
Chromatograms of the bicarbonate separation method confirming the identification of the separated As species based on the overlapping retention times of their peaks. The concentration of each As specie in the standards and in their mixed solution was 15 µg As l^−1^ (the original y scale applies to the TMAO chromatogram, other species signals have been shifted by increment of 5000 counts to improve the visual clarity. The shape of the As^III^ peak is magnified in the top right-hand corner of the figure)

Because of very low concentrations of As species in the PM_10_, we were looking for an easy and fast method for simultaneous separation of all five species with sufficient limits of detection. Therefore, in this work, a gradient elution HPLC separation method with ammonium bicarbonate MP and gradient elution was used for the separation of the As species. The advantage of this method is that it allows the separation of all five As species in only one separation step at one PRP-X100 chromatographic column. The identification of the As species peaks according to their retention times (RT) is shown in the chromatogram in Fig. 5. The disadvantage is the low sensitivity to the As^III^ species since the peak is not high and tails strongly. Therefore, higher detection limits are to be expected. Nevertheless, the obtained correlation factors of the As^III^ calibration curves, *R*, ranged between 0.9953 and 0.9999, the limits of detection varied between 0.10 and 0.89 µg As l^−1^, and the limits of quantification were in the range from 0.30 to 2.97 µg As l^−1^, with exact value depending on the daily performance of the instrument.

**Fig. 6:**
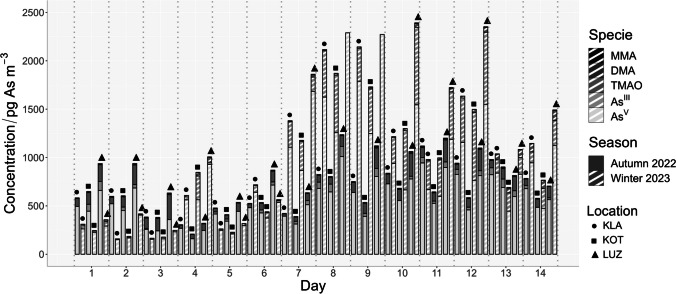
Results of As speciation analysis of all 84 samples from all three sampling locations and both sampling campaigns. Source data are available in the SI, Table S13

The results of the speciation analysis of all 84 samples from both sampling campaigns and all three sampling locations are shown in Fig. 6.

As is evident from the results summarised in Table 1, As^V^ was the major species in all the analysed PM_10_ samples and hence it follows the same seasonal and spatial trends as As_Total_ described above. It also did not show any seasonal trend in the proportional content (% representation; SI, Table S14 and Fig S6). The organic As species (As_org_) showed a clear visible seasonal dependence (Table 1). While the content of As_org_ was only 4% of the sum of all five As species in winter, it accounted for almost 17% in autumn (SI, Table S15 and Fig S7). The seasonal effect could be caused by several factors, one of them being temperature. Mukai et al. (1986) related the concentration of DMA and TMAO to the ambient temperature and estimated the activation energy of As biomethylation in nature to be approximately 12 kcal mol^−1^. Longer daytime with increased solar activity, and therefore more intensive UV light (Jakob et al. 2010), can lead to higher instability of volatile trivalent As species and their transformation into their pentavalent analogues, which can be than found in PM. Higher atmospheric humidity during the warmer period of the year could also play an important role. Tanda et al. (2019) were able to perform a speciation analysis of ELPI + size-resolved PM_x_ samples and found the highest concentrations of DMA and TMAO in particles from 0.15 to 0.5 µm. They hypothesised that the volatile As_org_ species are adsorbed on the surface of the accumulation mode particles, producing the MMA, DMA, and TMAO species. However, their study was performed in a relatively stable environment of a therapeutic cave. We did not speciate As in the PM_x_ samples, so, we can only logically hypothesise that in an outdoor environment with constantly changing meteorological conditions and variable humidity; the volatile As_org_ species could also be absorbed by the liquid phase of PM, made up of microscopic droplets, leaving higher concentrations of the As_org_ species also in the droplet mode particles. Future speciation analysis of PM_x_ samples might shed some light on this hypothesis. Last, but not least, a higher activity of the As_org_ producers (such as microorganisms, fungi) during the warmer months could be an important factor, since biovolatilisation has been already well documented (Bentley and Chasteen 2002; Cullen et al. 1995; Maeda et al. 1992; Cullen 2014; Ye et al. 2012). A decrease of As_org_ in PM_10_ during the winter season was also observed in studies of Mukai et al. (1986) performed in Oki Islands and Tsukuba, Japan, or by Xie et al. (2021) in Baoding, China. On the other hand, there are also studies that did not find any seasonal variations, for example, Tanda et al. (2020) in Austria, or Jakob et al. (2010) in Argentina. In the latter case, the sampling was performed at different sites, which may make it difficult to estimate seasonal changes. Tziaras et al. (2015) in Greece even observed an opposite trend. The TMAO concentrations they observed were clearly higher in autumn and winter compared to spring and summer, which could have been influenced by the island environment of the eastern Mediterranean, since biovolatilisation by microorganisms can also occur in seawater (Savage et al. 2018).

**Table 1:** Summarised results of the As speciation analysis in 84 PM_10_ samples, with seasonal and spatial dependency (*n* number of samples in which the species was detected, *n*_Tot_ total number of samples per location/season, % percentage content of As in the given species form compared to the sum of As in all analysed samples per the given location/season)

Location/season	Specie	Concentration (pg As m^−3^)	Proportional content (%)	Abundance (*n*/*n*_Tot_)
All/all	As^III^ As^V^ MMA DMA TMAO	133 ± 195 686 ± 448 1.69 ± 2.33 4.31 ± 4.46 66.4 ± 59.5	10.2 79.5 0.222 0.631 9.45	60/84 84/84 42/84 58/84 84/84
Seasonal dependency				
All/autumn 2022	As^III^ As^V^ MMA DMA TMAO	36.8 ± 31.3 581 ± 210 1.66 ± 2.62 8.36 ± 3.29 110 ± 60	4.02 79.3 0.199 1.20 15.3	28/42 42/42 17/42 42/42 42/42
All/winter 2023	As^III^ As^V^ MMA DMA TMAO	233 ± 235 816 ± 573 1.80 ± 2.18 0.569 ± 0.935 27.7 ± 24.6	16.3 79.8 0.246 0.058 3.63	32/42 42/42 25/42 16/42 42/42
Location dependency				
KLA/all	As^III^ As^V^ MMA DMA TMAO	100 ± 144 687 ± 401 0.75 ± 1.65 3.47 ± 3.55 44.3 ± 35.5	8.06 84.5 0.088 0.528 6.81	18/28 28/28 6/28 16/28 28/28
LUZ/all	As^III^ As^V^ MMA DMA TMAO	149 ± 224 866 ± 558 2.27 ± 2.86 6.00 ± 5.54 98.7 ± 79.8	8.83 79.0 0.196 0.715 11.3	22/28 28/28 15/28 27/28 28/28
KOT/all	As^III^ As^V^ MMA DMA TMAO	149 ± 211 505 ± 279 2.04 ± 2.12 3.45 ± 3.65 56.1 ± 39.6	13.6 75.1 0.383 0.650 10.3	20/28 28/28 21/28 15/28 28/28

In addition to seasonal trends, the effect of sampling location on As speciation was also monitored. Our location-related hypotheses focused primarily on the relationship between As_org_ content and the abundance of biomethylation producers in the vicinity, and vice versa the abundance of inorganic As species in the vicinity of potential emission sources inside the city. We decided to compare the proportional content (percentage representations) of the As species with their sum rather than their total concentration per location, because we were interested in the distribution of As species in PM, and As_Total_ was already discussed above. This way, not only the species concentrations in certain samples are considered, but also the abundance of samples containing these species.

The percentage of As_org_ in the air decreased at the sampling locations in the order LUZ (12.2%) > KOT (11.3%) > KLA (7.4). This outcome contradicts our biomethylation hypothesis, that locations rich in biomethylation producers would show increased content of As_org_. Also, the statistical comparison of sampling locations (SI, Table S16 and Fig. S8) did not prove the difference at a 0.05 significance level, and the hypothesis was therefore rejected. One of our hypotheses was that the proportional content of the inorganic As species in PM, As^III^, and As^V^, would be higher at the locations inside the city, closer to the primary emission sources such as traffic or household heating in winter. This turned out to be true only for As^III^, the proportional content of which decreased at the locations in the order KOT > LUZ > KLA. However, the difference between the locations did not prove to be significant at the 0.05 level (SI, Table S17 and S18, Fig. S9 and S10). A rather unexpected were the results for As^V^, where the order was exactly the opposite. In this case, the locations differed significantly at the 0.05 significance level (SI, Table S19 and Fig. S11). This is probably due to the extraction efficiency (described in SI, chapter Arsenic water extraction efficiency). As^V^ originates mainly from anthropogenic sources, and it is therefore probably located inside the larger diameter particles, which are more difficult to extract, whereas As_org_ becomes part of PM by adsorption of these volatile As_org_ species on the surface of already existing particles and its extraction is therefore much more efficient.

The concentrations and the abundance of the As_org_ species were generally decreased in the order TMAO > DMA > MMA, with only one exception which occurred at the KOT location, where MMA was present in more samples than DMA. This observation is consistent with previous publications (Mukai and Ambe 1987; Tanda et al. 2019, 2020; Tziaras et al. 2015; Xie et al. 2021). The fact that TMAO was present in all the samples analysed regardless of location or season supports the theory that TMAO is ubiquitous in PM.

## Conclusion

A new chromatographic method of As speciation was used in this work, including the separation of As species in PM_10_ water extracts at column PRP-X100 with ammonium bicarbonate buffer mobile phase and gradient elution, enabling simultaneous separation of all five analysed As species during one chromatographic analysis step.

The comparison of two sampling campaigns, carried out in the autumn of 2022 and in the winter of 2023, confirmed a seasonal influence on the content of As in PM_10_. The total As content in mineralised PM_10_ samples was higher during the winter due to seasonal changes in the PM_10_ sources, with household heating being the most likely source. The seasonal influence on PM composition was supported by the analysis of the As distribution in different PM_x_ size fractions. The results of speciation analysis showed an increase in the proportion of the As_org_ species in PM_10_ during the growing season (autumn campaign), when biovolatilisation producers are more active. On the contrary, the relation between the concentration of inorganic or methylated As species and the abundance of the biomethylation producers at the sampling location was not conclusive.

## Supplementary Information

Below is the link to the electronic supplementary material.

Supplementary file1 (DOCX 3230 KB)

## Data Availability

The datasets generated during and/or analysed during the current study are available from the corresponding author on reasonable request.

## Code availability—software application or custom code

The data were processed in the Origin SW as stated in the chapter  Statistical analysis. Any special software application or custom code wasn't used.
