# Supplementary material for: Seasonal and spatial variations of arsenic and its species in particulate matter in an urban environment of Brno, Czech Republic
Source: Environ Sci Pollut Res Int. 2024 Sep 3;31(43):55251–62. doi: 10.1007/s11356-024-34645-4 (PMC11415447; doi:10.1007/s11356-024-34645-4)
Supplement: Supplementary file 1 — Supplementary file1 (DOCX 3230 KB) [file 11356_2024_34645_MOESM1_ESM.docx]

# Supplementary Information

**Table S1** SW-4 Microwave program used for digestion of the filters and foils

| Step | Temperature  [ °C ] | Pressure  [ bar ] | Time  [ min ] | Power  [ W ] | Ramp  [ min ] |
| --- | --- | --- | --- | --- | --- |
| 1 | 130 | 25 | 8 | 60 | 5 |
| 2 | 180 | 30 | 15 | 60 | 3 |
| 3 | 230 | 30 | 25 | 60 | 3 |
| 4 | 50 | 5 | 10 | 0 | 1 |
| 5 | 50 | 1 | 12 | 0 | 1 |
| Notes:  Every sample was placed into an PTFE insert and filled with the mineralization mixture solution. Group of three inserts was then placed into DAK-100 digestion tube filled with 10 ml of ultrapure HNO_3_. | | | | | |

**Table S2** Trueness estimation of the total As analysis in digested PM samples based on bias from target value of simultaneously analysed reference materials

|  | Certified value | Observed value mean | Bias of Mean value | Number of replicates |
| --- | --- | --- | --- | --- |
| 1640a | 8.075 ± 0.070 µg As l^-1^ | 8.191 ± 0.088 µg As l^-1^ | 1.44 % | 4 |
| TM 35.2 | 7.02 ± 0.55 µg As l^-1^ | 7.289 ± 0.092 µg As l^-1^ | 3.84 % | 4 |


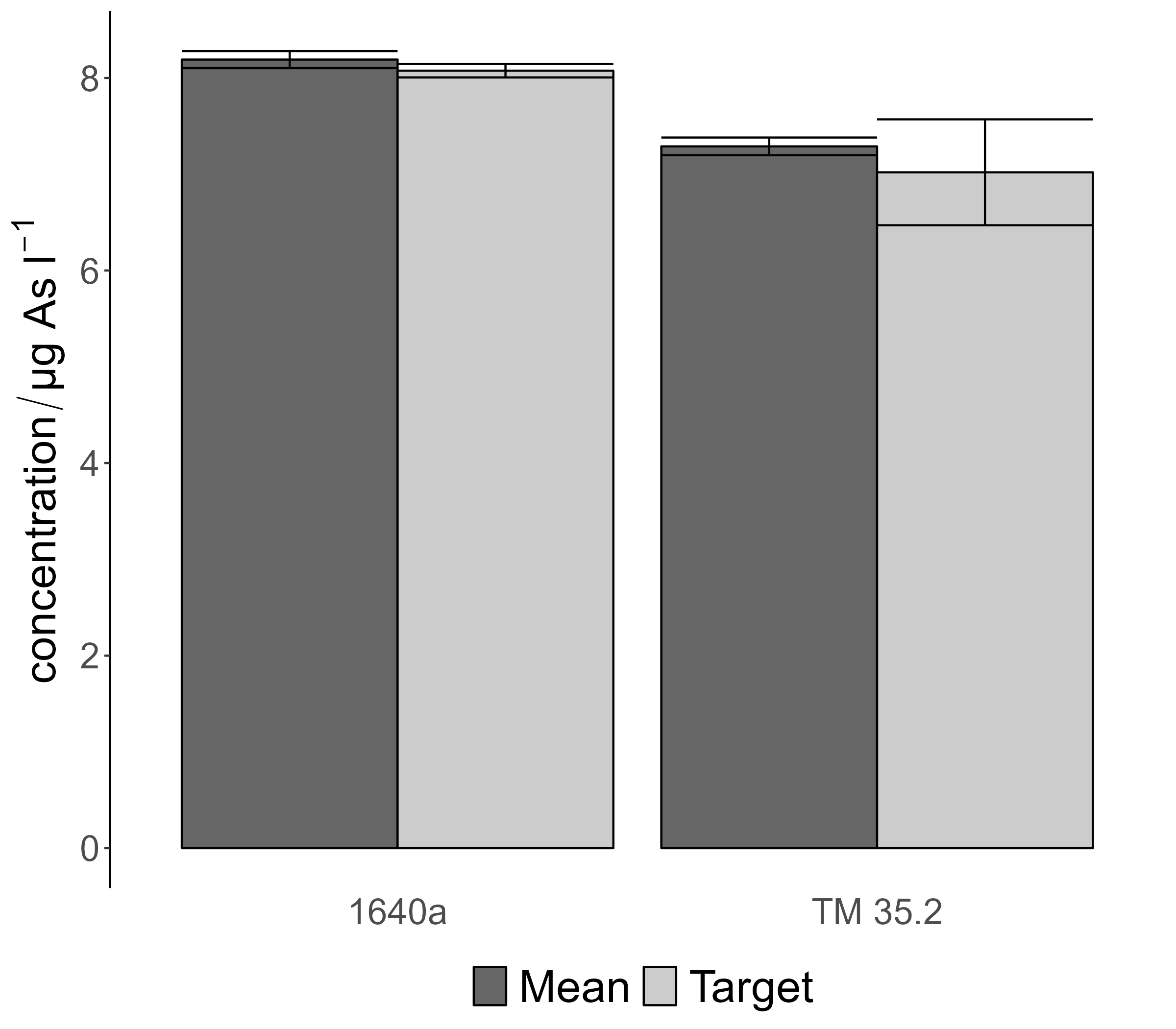


**Fig. S1** Trueness estimation of the total As analysis in digested PM samples based on bias from target value of simultaneously analysed reference materials. (Based on data in Table S1)

**Table S3** Limits of detection and quantification determined for the analysis of As total in mineralized PM samples

| LoD | LoQ |
| --- | --- |
| 0.0054 µg As l^-1^ | 0.014 µg As l^-1^ |

**Table S4** Gradient used for the HPLC separation of As species

| Time  [ min ] | 10 mM MF  [ % ] | 100 mM MF  [ % ] | methanol  [ % ] | Flow rate  [ ml/min ] |
| --- | --- | --- | --- | --- |
| 0 | 97 | 0 | 3 | 0.35 |
| 2.5 | 97 | 0 | 3 | 0.35 |
| 2.52 | 0 | 97 | 3 | 0.35 |
| 7.00 | 0 | 97 | 3 | 0.35 |
| 7.02 | 97 | 0 | 3 | 0.35 |

**Table S5** Total PM_10_ concentrations measured during both sampling campaigns at the three chosen localities in units of µg PM_10_ per m^3^ of filtered air. Expanded measurement uncertainty (confidence level approximately 95 %) was 0.1 µg m^-3^. Values exceeding the WHO recommended limit for 24-hour mean 45 µg m^-3^ (WHO, 2021) are marked by *

| Day | Locality | | | | | |
| --- | --- | --- | --- | --- | --- | --- |
|  | KLA | | LUZ | | KOT | |
|  | Campaign | | Campaign | | Campaign | |
|  | Autumn 2022 | Winter 2023 | Autumn 2022 | Winter 2023 | Autumn 2022 | Winter 2023 |
| 1 | 21.9 | 7.4 | 24.5 | 8.1 | 27.4 | 12.2 |
| 2 | 22.9 | 1.1 | 26.9 | 4.3 | 32.2 | 1.4 |
| 3 | 29.8 | 4.0 | 35.4 | 4.7 | 38.5 | 3.4 |
| 4 | 13.3 | 5.5 | 12.9 | 9.1 | 15.9 | 13.8 |
| 5 | 17.8 | 3.4 | 21.9 | 8.0 | 26.2 | 4.8 |
| 6 | 23.5 | 1.7 | 27.8 | 6.2 | 33.9 | 5.0 |
| 7 | 19.4 | 7.2 | 21.9 | 17.2 | 27.0 | 10.2 |
| 8 | 44.6 | 24.6 | 40.8 | 35.3 | 47.8* | 35.7 |
| 9 | 42.6 | 20.9 | 41.7 | 32.7 | 47.6* | 33.9 |
| 10 | 34.7 | 21.7 | 35.5 | 37.3 | 39.1 | 36.0 |
| 11 | 35.8 | 31.3 | 33.3 | 37.1 | 37.6 | 37.6 |
| 12 | 27.7 | 34.6 | 31.6 | 51.7* | 36.8 | 61.6* |
| 13 | 36.2 | 21.2 | 38.4 | 29.0 | 43.5 | 27.8 |
| 14 | 14.1 | 8.4 | 16.8 | 5.5 | 22.5 | 9.8 |

***Table S6*** *Content of PM_10_ in the air - comparison between seasons*

| **Input Data** | | | | | | | | | | |
| --- | --- | --- | --- | --- | --- | --- | --- | --- | --- | --- |
|  | | | | | Data | | | | Range | |
| Dependent Variable | | | | | PM_10_ [µg m^-3^] | | | | [1*:84*] | |
| Factor | | | | | Campaign | | | | [1*:84*] | |
| **Descriptive Statistics** | | | | | | | | | | |
|  | | N Analysis | | N Missing | | Mean | | Standard Deviation | | SE of Mean |
| Autumn 2022 | | 41 | | 0 | | 30.55854 | | 9.55026 | | 1.4915 |
| Winter 2023 | | 43 | | 0 | | 18.35349 | | 15.19098 | | 2.3166 |
| **One Way ANOVA** | | | | | | | | | | |
|  | DF | | Sum of Squares | | | | Mean Square | | F Value | Prob>F |
| Model | 1 | | 3126.45387 | | | | 3126.45387 | | 19.21741 | <0.0001 |
| Error | 82 | | 13340.46649 | | | | 162.68862 | |  |  |
| Total | 83 | | 16466.92036 | | | |  | |  |  |
| Null Hypothesis: The Means of all levels are equal.  Alternative Hypothesis: The means of one or more levels are different.  At the 0.05 level, the population means are significantly different. | | | | | | | | | | |


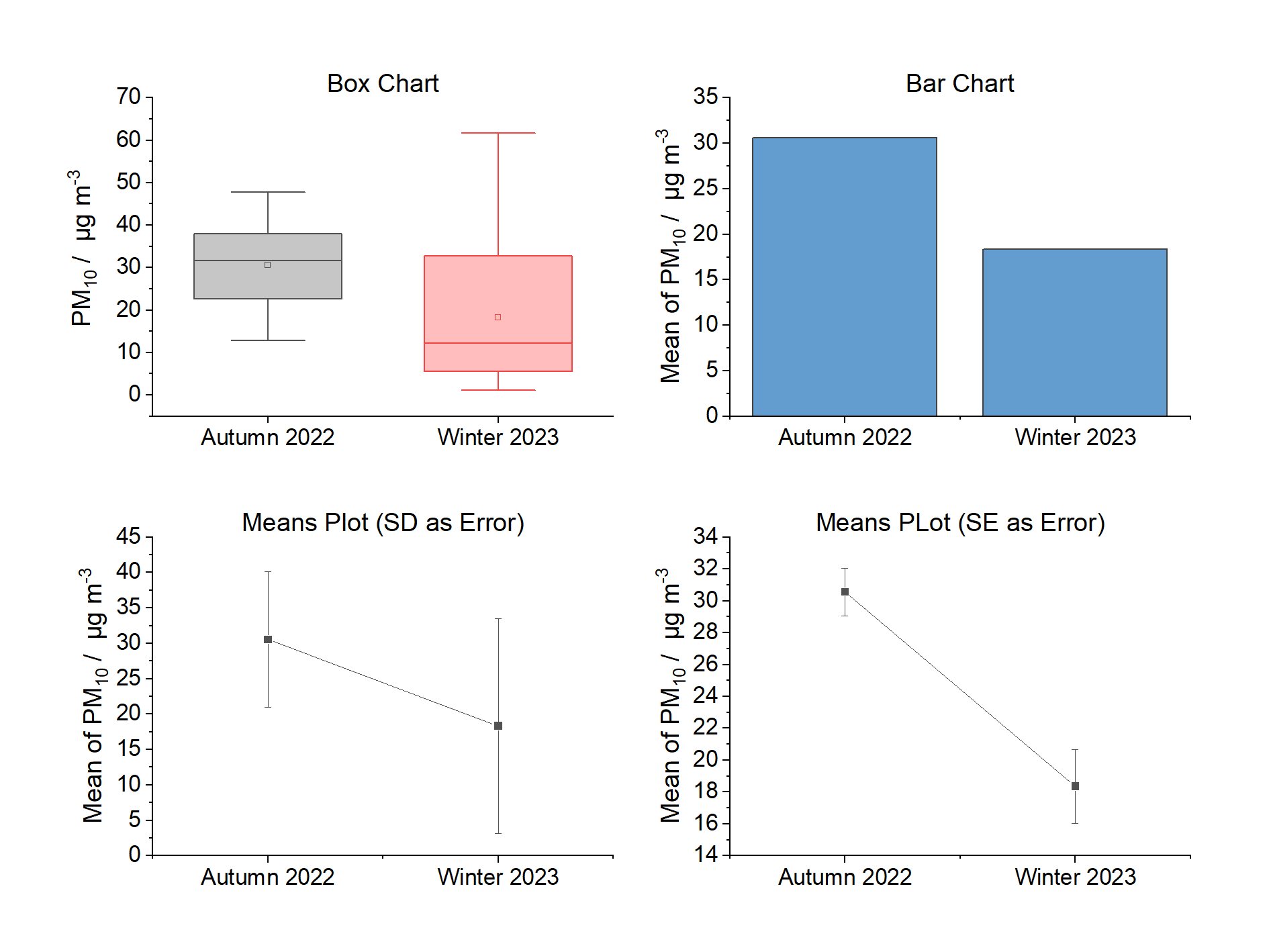


**Fig. S2** Content of PM_10_ in the air. At the 0.05 level, population means differ significantly between seasons

**Table S7** As_Total_ concentrations in mineralized PM_10_ samples collected during both sampling campaigns for the three chosen localities in units of pg As per m^3^ of filtered air

| Day | Locality | | | | | |
| --- | --- | --- | --- | --- | --- | --- |
|  | KLA | | LUZ | | KOT | |
|  | Campaign | | Campaign | | Campaign | |
|  | Autumn 2022 | Winter 2023 | Autumn 2022 | Winter 2023 | Autumn 2022 | Winter 2023 |
| 1 | 574 ± 15 | 489 ± 15 | 985 ± 30 | 398 ± 12 | 824 ± 21 | 428 ± 15 |
| 2 | 634 ± 18 | 291 ± 19 | 1002 ± 27 | 262 ± 15 | 946 ± 15 | 263 ± 14 |
| 3 | 449 ± 20 | 182 ± 16 | 616 ± 14 | 240.0 ± 8.9 | 524 ± 23 | 270 ± 17 |
| 4 | 350.1 ± 7.8 | 788 ± 41 | 313 ± 22 | 1399 ± 33 | 350 ± 10 | 1521 ± 81 |
| 5 | 524 ± 10 | 326 ± 11 | 665 ± 14 | 303 ± 14 | 639 ± 24 | 346 ± 18 |
| 6 | 677 ± 27 | 830 ± 31 | 970 ± 15 | 677 ± 34 | 791 ± 14 | 632 ± 26 |
| 7 | 671 ± 26 | 1502 ± 42 | 779 ± 28 | 2039 ± 37 | 773 ± 11 | 1675 ± 17 |
| 8 | 1143 ± 19 | 1723 ± 42 | 1366 ± 34 | 3050 ± 36 | 1350 ± 18 | 2741 ± 75 |
| 9 | 877 ± 27 | 1683 ± 49 | 1065 ± 34 | 3209 ± 95 | 1109 ± 27 | 2273 ± 38 |
| 10 | 982 ± 33 | 972 ± 22 | 972.9 ± 9.1 | 2483 ± 61 | 1011 ± 21 | 1819 ± 36 |
| 11 | 1048 ± 18 | 748 ± 24 | 1173 ± 12 | 1819 ± 45 | 1223 ± 100 | 1184 ± 47 |
| 12 | 907 ± 17 | 1802 ± 64 | 1195 ± 38 | 2952 ± 80 | 1006 ± 22 | 2220 ± 34 |
| 13 | 892 ± 23 | 1167 ± 43 | 1060 ± 11 | 1364 ± 41 | 988 ± 17 | 1282 ± 49 |
| 14 | 752 ± 14 | 856.2 ± 8.2 | 828 ± 30 | 1998 ± 56 | 781 ± 19 | 1640 ± 34 |

**Table S8** As_Total_ concentrations in mineralized PM_10_ samples collected during both sampling campaigns for the three chosen localities in units of ng As per mg of PM_10_

| Day | Locality | | | | | |
| --- | --- | --- | --- | --- | --- | --- |
|  | KLA | | LUZ | | KOT | |
|  | Campaign | | Campaign | | Campaign | |
|  | Autumn 2022 | Winter 2023 | Autumn 2022 | Winter 2023 | Autumn 2022 | Winter 2023 |
| 1 | 26.20 ± 0.68 | 65.9 ± 2.0 | 40.2 ± 1.2 | 49.4 ± 1.5 | 30.11 ± 0.77 | 35.1 ± 1.2 |
| 2 | 27.71 ± 0.79 | 263 ± 18 | 37.3 ± 1.0 | 61.0 ± 3.6 | 29.37 ± 0.47 | 186.0 ± 9.9 |
| 3 | 15.06 ± 0.67 | 45.4 ± 4.0 | 17.40 ± 0.40 | 51.2 ± 1.9 | 13.62 ± 0.59 | 79.5 ± 5.0 |
| 4 | 26.41 ± 0.59 | 142.9 ± 7.5 | 24.4 ± 1.7 | 153.0 ± 3.6 | 22.01 ± 0.64 | 110.1 ± 5.9 |
| 5 | 29.49 ± 0.57 | 95.4 ± 3.1 | 30.36 ± 0.65 | 37.8 ± 1.7 | 24.36 ± 0.90 | 72.5 ± 3.7 |
| 6 | 28.9 ± 1.1 | 492 ± 19 | 34.88 ± 0.56 | 109.2 ± 5.4 | 23.31 ± 0.41 | 126.8 ± 5.2 |
| 7 | 34.5 ± 1.3 | 208.6 ± 5.9 | 35.6 ± 1.3 | 121.9 ± 2.1 | 28.65 ± 0.41 | 163.6 ± 1.7 |
| 8 | 25.59 ± 0.43 | 70.2 ± 1.7 | 33.48 ± 0.83 | 86.3 ± 1.0 | 28.27 ± 0.37 | 76.7 ± 2.1 |
| 9 | 20.58 ± 0.63 | 80.4 ± 2.3 | 25.54 ± 0.82 | 98.1 ± 2.9 | 23.32 ± 0.57 | 67.0 ± 1.1 |
| 10 | 28.31 ± 0.96 | 44.8 ± 1.0 | 27.38 ± 0.26 | 66.6 ± 1.6 | 25.85 ± 0.54 | 50.6 ± 1.0 |
| 11 | 29.26 ± 0.49 | 23.90 ± 0.77 | 35.22 ± 0.36 | 49.0 ± 1.2 | 32.5 ± 2.7 | 31.5 ± 1.3 |
| 12 | 32.81 ± 0.61 | 52.1 ± 1.8 | 37.8 ± 1.2 | 57.1 ± 1.6 | 27.35 ± 0.59 | 36.06 ± 0.56 |
| 13 | 24.64 ± 0.63 | 55.0 ± 2.0 | 27.63 ± 0.29 | 47.0 ± 1.4 | 22.68 ± 0.38 | 46.2 ± 1.8 |
| 14 | 53.2 ± 1.0 | 101.36 ± 0.97 | 49.3 ± 1.8 | 365 ± 10 | 34.64 ± 0.84 | 167.0 ± 3.5 |

***Table S9*** *Content of As_Total_ in the mineralized samples of PM_10_ - comparison between seasons*

| **Input Data** | | | | | |
| --- | --- | --- | --- | --- | --- |
|  | | Data | | Range | |
| Dependent Variable | | As_Total_ [ng As mg^-1^] | | [1*:84*] | |
| Factor | | Campaign | | [1*:84*] | |
| **Descriptive Statistics** | | | | | |
|  | N Analysis | N Missing | Mean | Standard Deviation | SE of Mean |
| Autumn 2022 | 42 | 0 | 29.17119 | 7.6622 | 1.1823 |
| Winter 2023 | 42 | 0 | 103.38619 | 91.3791 | 14.1001 |
| **One Way ANOVA** | | | | | |
|  | DF | Sum of Squares | Mean Square | F Value | Prob>F |
| Model | 1 | 115665.19072 | 115665.19072 | 27.51035 | <0.0001 |
| Error | 82 | 344762.84783 | 4204.42497 |  |  |
| Total | 83 | 460428.03856 |  |  |  |
| Null Hypothesis: The Means of all levels are equal.  Alternative Hypothesis: The means of one or more levels are different.  At the 0.05 level, the population means are significantly different. | | | | | |


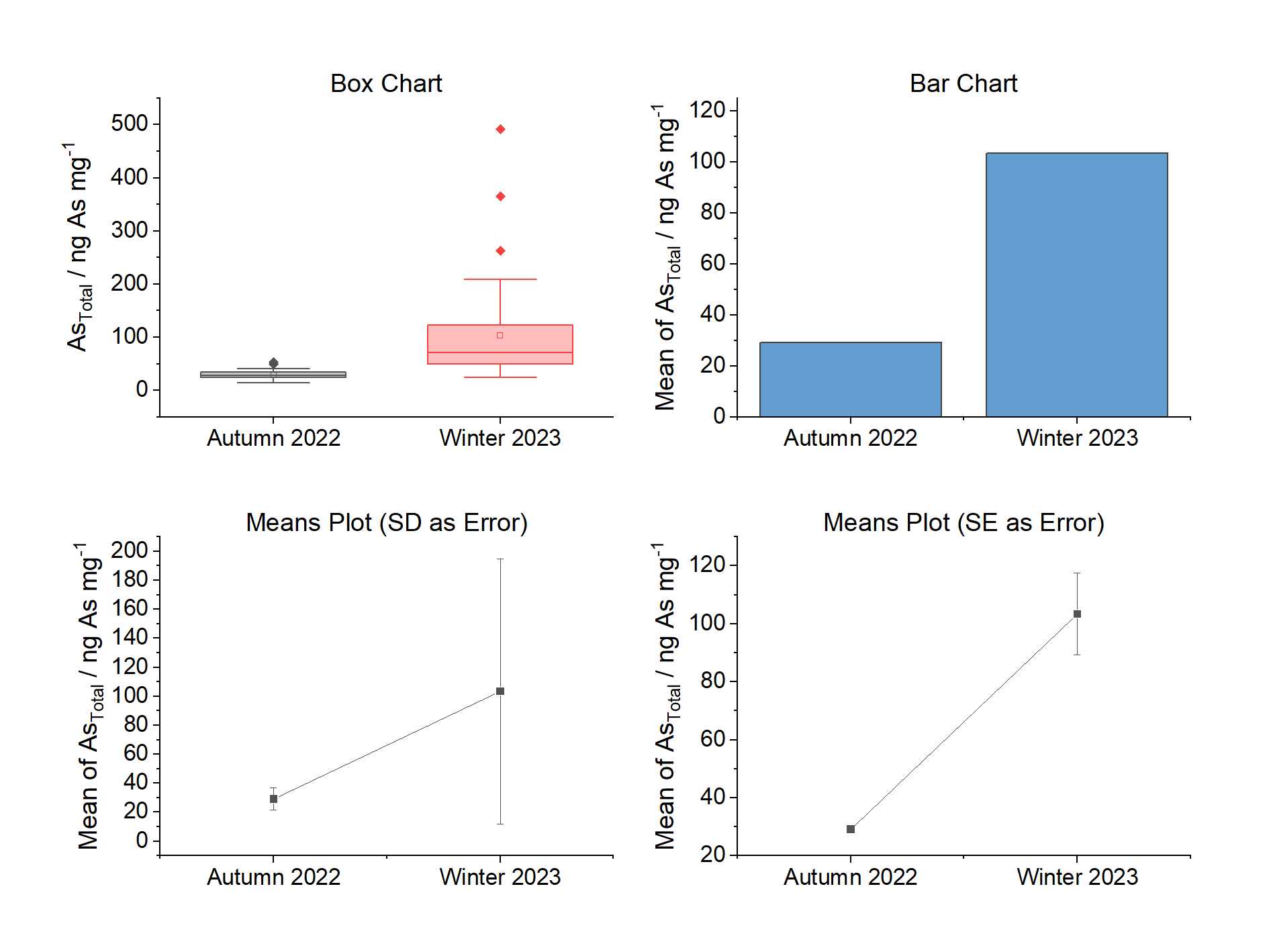


**Fig. S3** Content of As_Total_ in the mineralized samples of PM_10_. At the 0.05 level, population means differ significantly between seasons

**Table S10** Content of As_Total_ in the mineralized samples of PM_10_ - comparison between locations

| **Input Data** | | | | | |
| --- | --- | --- | --- | --- | --- |
|  | | Data | | Range | |
| Dependent Variable | | As_Total_ [pg As m^-3^] | | [1*:84*] | |
| Factor | | Location | | [1*:84*] | |
| **Descriptive Statistics** | | | | | |
|  | N Analysis | N Missing | Mean | Standard Deviation | SE of Mean |
| KLA | 28 | 0 | 851.40357 | 427.76916 | 80.84077 |
| KOT | 28 | 0 | 1093.17857 | 636.10441 | 120.21243 |
| LUZ | 28 | 0 | 1256.53214 | 847.29573 | 160.12384 |
| **One Way ANOVA** | | | | | |
|  | DF | Sum of Squares | Mean Square | F Value | Prob>F |
| Model | 2 | 2326507.86024 | 1163253.93012 | 2.67307 | 0.07513 |
| Error | 81 | 3.52492E7 | 435175.11207 |  |  |
| Total | 83 | 3.75757E7 |  |  |  |
| Null Hypothesis: The Means of all levels are equal.  Alternative Hypothesis: The means of one or more levels are different.  At the 0.05 level, the population means are not significantly different. | | | | | |


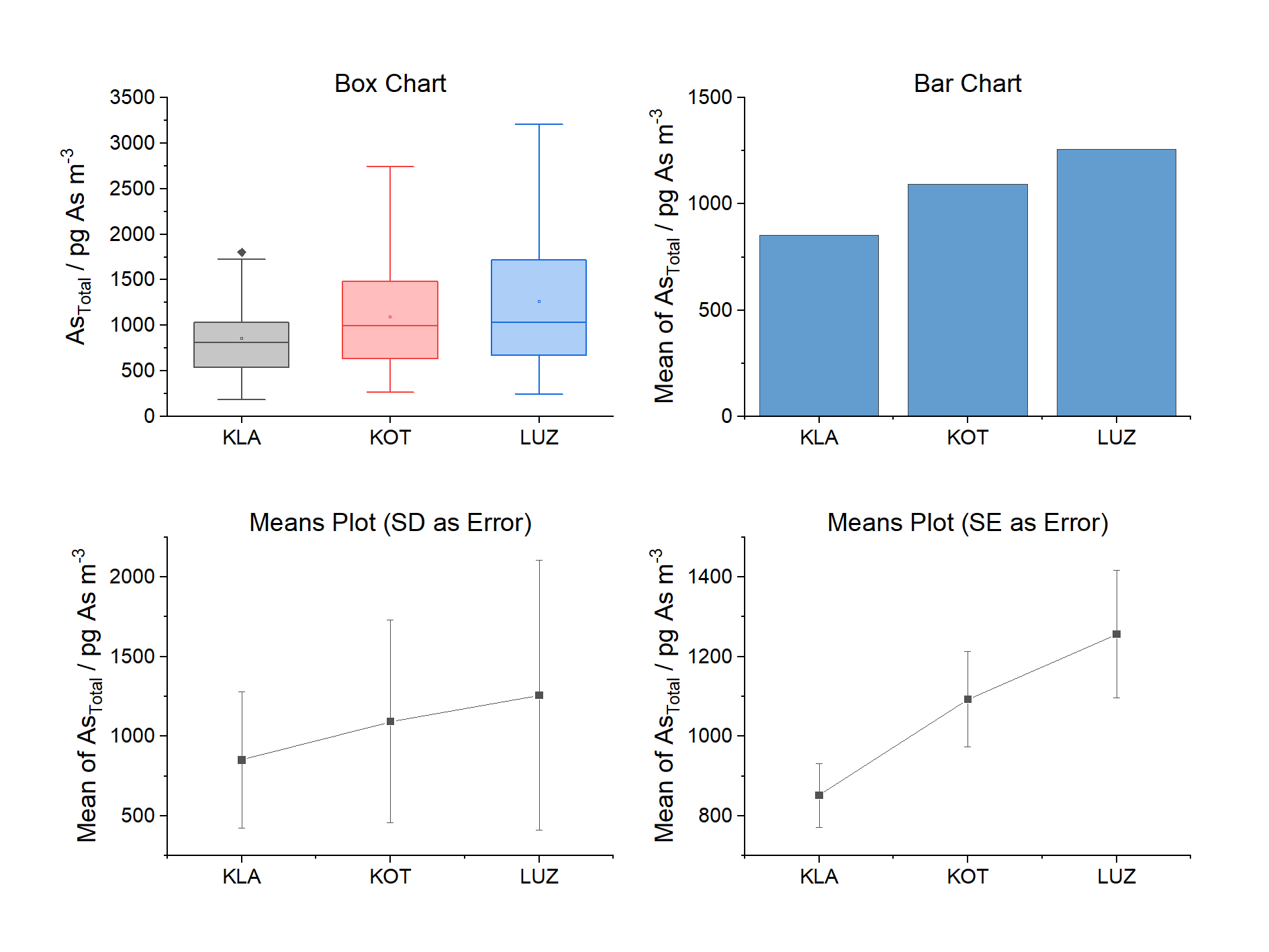


**Fig. S4** Content of As_Total_ in the mineralized samples of PM_10_. At the 0.05 level, population means don’t differ significantly between locations


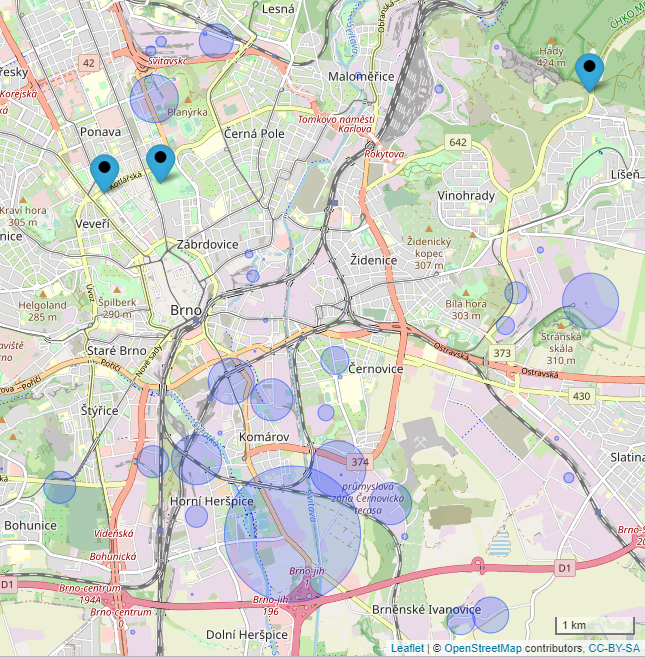


**Fig. S5** Sampling locations (markers) and positions of major stationary sources of PM (circles) in Brno city. Based on CHMI (2022) data (Table S11). Size of the circles represents potential of each source (tons of PM emissions per year 2022), not their reach. (Cheng J et al., 2022; OpenStreetMap)

**Table S11** Source data for Fig. S2. Location closest to the sampling points inside the city is in bold letters. Portable and moving equipment isn’t included in this list

| **latitude** | **longitude** | **PM / year 2022** | **Source (company)** |
| --- | --- | --- | --- |
| 49.12819 | 16.65916 | 0.049 t | NAVOS a.s. - NS Chrlice |
| 49.12838 | 16.65658 | 0.015 t | ALFE BRNO s.r.o. - slévárna |
| 49.12839 | 16.67787 | 0.083 t | HASIT Šumavské vápenice a omítkárny s.r.o. - Brno-Chrlice |
| 49.12962 | 16.67842 | 0.269 t | Brněnská obalovna s.r.o. - obalovna Chrlice |
| 49.13461 | 16.67104 | 0.139 t | Krematorium zvířat Brno s.r.o. - Brno |
| 49.13678 | 16.66705 | 0.228 t | Smurfit Kappa Czech |
| 49.14039 | 16.60219 | 1.083 t | TRANSBETON s.r.o. |
| 49.14345 | 16.60328 | 0.889 t | REMET spol. s r.o. - provoz Brno |
| 49.14458 | 16.60518 | 0.239 t | ZAPA UNISTAV s.r.o. - Brno-jih-Přízřenice |
| 49.1461 | 16.66846 | 0.033 t | Moravia Tech a.s. |
| 49.1517 | 16.60458 | 0.013 t | AB KOMPONENTY s.r.o. |
| 49.1561 | 16.60219 | 0.015 t |  |
| 49.15738 | 16.65855 | 0.021 t  0.753 t | Brněnská obalovna s.r.o. - obalovna Brněnské Ivanovice FRISCHBETON s.r.o. - betonárna Brněnské Ivanovice Brno-Tuřany |
| 49.15802 | 16.66363 | 1.212 t | RECYKLACE - PROCHÁZKA .s.r.o. |
| 49.16033 | 16.61239 | 0.001 t | ESB Rozvaděče a.s. |
| 49.16547 | 16.61368 | 0.007 t | SAND CORES; KM JADERNA s.r.o. - VÝROBA JADER |
| 49.16682 | 16.64888 | 0.047 t | SUEZ CZ a.s. - centrální kompostárna Brno |
| 49.16711 | 16.62961 | 17.822 t | Eligo a.s. - odštěpný závod Brno |
| 49.1691 | 16.61313 | 0.514 t | Skanska Transbeton s.r.o. - betonárna Brno-jih |
| 49.17052 | 16.64645 | 1.283 t  0.368 t | TBG BETONMIX a.s. - Betonárna Brno-Černovice Sievert CZ k.s. |
| 49.17236 | 16.58966 | 1.040 t | Pohřební a hřbitovní služby a.s. - provozovna Brno |
| 49.17323 | 16.54371 | 1.281 t | TBG BETONMIX a.s. - Betonárna Brno-Bosonohy |
| 49.17343 | 16.67706 | 0.008 t  0.001 t | stoba Precizní Technika s.r.o. Atlas Copco Services s.r.o. |
| 49.17375 | 16.63797 | 0.001 t | Povrchové úpravy Brno s.r.o. - Brno - Černovice |
| 49.17441 | 16.6374 | 3.132 t | SETRA spol. s r. o. - recyklace Brno-Černovice |
| 49.17443 | 16.57969 | 0.003 t | Vězeňská služba České republiky - vazební věznice Brno-Bohunice |
| 49.17523 | 16.60564 | 0.965 t | STAPPA mix Brno - provozovna Brno |
| 49.17546 | 16.61313 | 2.444 t | DRAXTON BRNO s.r.o. - slévárna |
| 49.17785 | 16.67496 | 0.009 t | Porsche Inter Auto CZ spol. s r.o. - Brno-Slatina |
| 49.17844 | 16.61718 | 0.018 t | CHRIŠTOF spol. s r.o. provoz Brno |
| 49.18072 | 16.63536 | 0.234 t | Nová Mosilana a.s. |
| 49.18218 | 16.62606 | 1.693 t | CEMEX Czech Republic s.r.o. - betonárna Brno |
| 49.18426 | 16.61909 | 2.126 t | UXA spol. s r.o. |
| 49.18473 | 16.63777 | 0.016 t | PSYCHIATRICKÁ LÉČEBNA |
| 49.1866 | 16.63688 | 0.761 t | Pískovna Černovice spol. s r.o. |
| 49.18762 | 16.62314 | 0.005 t | Autonova Brno spol. s r.o. - Brno-střed |
| 49.19051 | 16.66616 | 0.326 t | SAKO Brno a.s.- divize 3 ZEVO |
| 49.19108 | 16.625 | 0.012 t | Krajské ředitelství policie Jihomoravského kraje - Křenová Brno-střed |
| 49.19325 | 16.68078 | 2.992 t | Slévárna HEUNISCH Brno s.r.o. |
| 49.19417 | 16.66795 | 0.459 t | Kalcit s.r.o. - lom Brno-Líšeň |
| 49.19605 | 16.62283 | 0.159 t | Teplárny Brno a.s. - Provoz Špitálka |
| 49.19792 | 16.62464 | 0.003 t | LARS Chemie spol. s r.o. |
| 49.19855 | 16.62215 | 0.050 t | BMT Medical Technology s.r.o. - Brno-střed |
| 49.19899 | 16.67217 | 0.046 t | ZETOR TRACTORS a.s. |
| 49.21558 | 16.50129 | 0.394 t | Teplárny Brno a.s. - Teyschlova 33 |
| **49.21596** | **16.60588** | **2.192 t** | **Teplárny Brno a.s.** |
| 49.21703 | 16.65477 | 0.064 t | PROCOMETAL - KOMEXPO s.r.o. |
| 49.21811 | 16.60936 | 0.005 t | VAKABRNOCZ s.r.o. - Cimburkova |
| 49.21857 | 16.6409 | 0.047 t | HEMA puls |
| 49.2213 | 16.6053 | 0.091 t | KRÁLOVOPOLSKÁ SLÉVÁRNA s.r.o. |
| 49.22172 | 16.66465 | 0.015 t | KROMA - Jaroslav Králík s.r.o. - Tryskače |
| 49.22235 | 16.60706 | 0.116 t | KRÁLOVOPOLSKÁ a.s. |
| 49.22242 | 16.61653 | 1.174 t | TBG BETONMIX a.s. - Betonárna Brno - Královo Pole |
| 49.22325 | 16.61457 | 0.012 t  0.002 t | FIRESTA-Fišer rekonstrukce stavby a.s. KPS KRÁLOVOPOLSKÁ KOVÁRNA s.r.o. |
| 49.22821 | 16.53557 | 0.480 t | Lesy Města Brna a.s. - Pila Bystrc |
| 49.23552 | 16.58527 | 0.135 t | Dopravní podnik města Brna a.s. - Hudcova Brno-Medlánky |
| 49.25141 | 16.5922 | 0.001 t | VUAB Pharma a.s. Cytostatické substance |
| 49.25327 | 16.59191 | 0.001 t | Erba Lachema s.r.o. |
| 49.25854 | 16.58094 | 0.004 t | MOTORTEC spol. s r.o. - Maříkova Brno-Ivanovice |

**Table S12** As_Total_ concentrations in mineralized PM_x_ samples collected during both sampling campaigns in units of units pg As per m^3^ of filtered air

| Size fraction | Campaign | |
| --- | --- | --- |
| [µm] | Autumn 2022 | Winter 2023 |
| 0.016 | 0.58 ± 0.30* | 5.69 ± 0.87 |
| 0.030 | 1.82 ± 0.48 | 1.91 ± 0.54 |
| 0.054 | 7.08 ± 0.33 | 6.0 ± 1.1 |
| 0.094 | 19.8 ± 1.4 | 9.55 ± 0.63 |
| 0.15 | 43.1 ± 1.7 | 13.40 ± 0.95 |
| 0.25 | 67.2 ± 2.2 | 23.7 ± 2.1 |
| 0.38 | 114.6 ± 3.3 | 202.9 ± 3.7 |
| 0.60 | 57.2 ± 1.9 | 244.2 ± 3.4 |
| 0.94 | 18.3 ± 1.1 | 93.8 ± 3.8 |
| 1.6 | 10.32 ± 0.80 | 38.7 ± 1.6 |
| 2.5 | 6.96 ± 0.61 | 27.2 ± 1.7 |
| 3.6 | 3.54 ± 0.33 | 6.1 ± 1.1 |
| 5.3 | 0.95 ± 0.73 | 2.10 ± 0.54 |
| 10 | 3.02 ± 0.48 | 0.49 ± 0.13* |
| * < LoQ ˄ > LoD | | |

**Table S13** Results of the As speciation analysis (Part I)

| As^III^ | | | | | | |
| --- | --- | --- | --- | --- | --- | --- |
| Day | Locality | | | | | |
|  | KLA | | LUZ | | KOT | |
|  | Campaign | | Campaign | | Campaign | |
|  | Autumn 2022 | Winter 2023 | Autumn 2022 | Winter 2023 | Autumn 2022 | Winter 2023 |
| 1 | < LoD | 39.4* | 93.8* | 45.8 | 71.7* | < LoD |
| 2 | < LoD | < LoD | 35.1* | < LoD | 35.9* | < LoD |
| 3 | 10.1* | < LoD | < LoD | < LoD | < LoD | < LoD |
| 4 | < LoD | 30.3* | < LoD | 23.2 | < LoD | 245 |
| 5 | 21.8* | < LoD | < LoD | < LoD | 22.7* | < LoD |
| 6 | < LoD | 71.7* | 32.1* | 19.6 | 30.7* | 56.5* |
| 7 | < LoD | 261 | 42.1* | 149 | 27.2* | 298* |
| 8 | 65.0* | 478 | 106* | 449 | 80.6* | 596 |
| 9 | < LoD | 342* | 83.7 | 422 | 31.7* | 461* |
| 10 | < LoD | 266* | 27.9 | 800 | < LoD | 611 |
| 11 | 56.7* | 298 | 57.6 | 490 | 23.7* | 376 |
| 12 | 52.9* | 459 | 50.7 | 745 | 28.2* | 698 |
| 13 | 71.6* | 136* | 60.0 | 173 | 87.5* | 188 |
| 14 | 32.4* | 113* | 18.8* | 259 | < LoD | 203 |
| As^V^ | | | | | | |
| Day | Locality | | | | | |
|  | KLA | | LUZ | | KOT | |
|  | Campaign | | Campaign | | Campaign | |
|  | Autumn 2022 | Winter 2023 | Autumn 2022 | Winter 2023 | Autumn 2022 | Winter 2023 |
| 1 | 494 | 262 | 659 | 292 | 445 | 226 |
| 2 | 522 | 151 | 685 | 405 | 452 | 169 |
| 3 | 257 | 154 | 358 | 232 | 242 | 160 |
| 4 | 268 | 563 | 246 | 929 | 160 | 563 |
| 5 | 416 | 248 | 443 | 296 | 342 | 210 |
| 6 | 482 | 641 | 716 | 535 | 427 | 375 |
| 7 | 394 | 1107 | 512 | 1683 | 313 | 866 |
| 8 | 681 | 1625 | 1010 | 2289 | 644 | 1256 |
| 9 | 638 | 1788 | 804 | 2272 | 391 | 1248 |
| 10 | 728 | 939 | 778 | 1545 | 555 | 665 |
| 11 | 944 | 668 | 897 | 1188 | 526 | 599 |
| 12 | 825 | 1156 | 813 | 1550 | 458 | 765 |
| 13 | 822 | 839 | 597 | 827 | 690 | 446 |
| 14 | 678 | 945 | 564 | 1122 | 485 | 475 |

**Table S13** Results of the As speciation analysis (Part II)

| MMA | | | | | | |
| --- | --- | --- | --- | --- | --- | --- |
| Day | Locality | | | | | |
|  | KLA | | LUZ | | KOT | |
|  | Campaign | | Campaign | | Campaign | |
|  | Autumn 2022 | Winter 2023 | Autumn 2022 | Winter 2023 | Autumn 2022 | Winter 2023 |
| 1 | < LoD | < LoD | < LoD | 3.72 | 5.69 | 5.37 |
| 2 | 2.71* | < LoD | < LoD | 2.08 | 2.36* | 2.20* |
| 3 | 1.98 | < LoD | < LoD | < LoD | 2.51* | 1.55* |
| 4 | < LoD | < LoD | < LoD | 1.02* | < LoD | 1.48* |
| 5 | < LoD | < LoD | < LoD | 1.12* | < LoD | 0.12 |
| 6 | < LoD | < LoD | < LoD | 1.05* | < LoD | 1.70* |
| 7 | < LoD | 3.21 | < LoD | 4.02 | < LoD | 1.90 |
| 8 | < LoD | < LoD | < LoD | 7.50 | < LoD | 2.42 |
| 9 | < LoD | < LoD | 10.3 | 5.73 | < LoD | 2.94 |
| 10 | < LoD | < LoD | 3.69* | 6.90 | 2.41* | 1.53 |
| 11 | 6.85 | < LoD | 3.43* | 4.47 | 1.74* | 0.73 |
| 12 | 4.02* | < LoD | 3.07* | 5.35 | 1.50* | 1.72 |
| 13 | 2.18* | < LoD | < LoD | < LoD | 8.80 | 5.59* |
| 14 | < LoD | < LoD | < LoD | < LoD | 2.92* | < LoD |
| DMA | | | | | | |
| Day | Locality | | | | | |
|  | KLA | | LUZ | | KOT | |
|  | Campaign | | Campaign | | Campaign | |
|  | Autumn 2022 | Winter 2023 | Autumn 2022 | Winter 2023 | Autumn 2022 | Winter 2023 |
| 1 | 6.53 | < LoD | 11.8 | < LoD | 6.45 | < LoD |
| 2 | 6.09 | < LoD | 14.0 | 0.44* | 8.08 | < LoD |
| 3 | 8.69 | < LoD | 16.6 | 0.75 | 10.1 | < LoD |
| 4 | 3.39* | < LoD | 5.76 | 1.79 | 3.78 | < LoD |
| 5 | 3.66 | < LoD | 6.46 | 0.72 | 3.56* | < LoD |
| 6 | 5.12 | < LoD | 9.74 | 0.56* | 6.13 | < LoD |
| 7 | 4.19 | < LoD | 6.80 | 0.63* | 4.95 | < LoD |
| 8 | 6.96 | < LoD | 9.54 | 0.76 | 5.19 | < LoD |
| 9 | 6.64 | < LoD | 11.3 | 0.68 | 7.16 | < LoD |
| 10 | 8.22 | < LoD | 14.3 | 1.16 | 9.60 | < LoD |
| 11 | 10.9 | < LoD | 15.1 | 1.84 | 7.77 | < LoD |
| 12 | 7.47 | < LoD | 12.8 | 1.35 | 7.92 | < LoD |
| 13 | 8.55 | 2.10* | 11.3 | 2.19 | 8.33 | < LoD |
| 14 | 5.82 | 2.78* | 5.85 | 3.72 | 5.26 | 2.43* |

**Table S13** Results of the As speciation analysis (Part III)

| TMAO | | | | | | | | |
| --- | --- | --- | --- | --- | --- | --- | --- | --- |
| Day | Locality | | | | | | | |
|  | KLA | | | LUZ | | | KOT | |
|  | Campaign | | | Campaign | | | Campaign | |
|  | Autumn 2022 | Winter 2023 | | Autumn 2022 | Winter 2023 | | Autumn 2022 | Winter 2023 |
| 1 | 81.4 | 8.93 | | 173 | 14.9 | | 120 | 14.0 |
| 2 | 67.5 | 7.41 | | 203 | 9.5 | | 106 | 13.5 |
| 3 | 107 | 10.9 | | 258 | 13.9 | | 127 | 16.3 |
| 4 | 33.1 | 14.9 | | 68.2 | 51.2 | | 43.9 | 37.1 |
| 5 | 35.7 | 13.5 | | 86.0 | 19.7 | | 40.6 | 17.1 |
| 6 | 38.5 | 5.02 | | 112 | 7.0 | | 70.3 | 6.0 |
| 7 | 23.7 | 8.67 | | 73.6 | 24.7 | | 44.2 | 11.8 |
| 8 | 71.1 | 12.0 | | 111 | 32.1 | | 72.0 | 15.9 |
| 9 | 106 | 12.8 | | 211 | 34.9 | | 105 | 18.5 |
| 10 | 101 | 10.8 | | 237 | 39.1 | | 115 | 21.0 |
| 11 | 101 | 13.1 | | 227 | 38.2 | | 85.5 | 19.4 |
| 12 | 52.1 | 18.8 | | 219 | 49.6 | | 88.8 | 33.8 |
| 13 | 74.4 | 59.8 | | 146 | 81.9 | | 107 | 50.6 |
| 14 | 67.0 | 85.3 | | 115 | 106 | | 85.1 | 84.9 |
| * > LoD ˄ < LoQ  Note: LoD and LoQ were calculated daily to correspond to the analyser’s performance. Therefore, the limits can differ from day to day.  For calculation were used formulas:  $LoD=\frac{3\cdot s}{b}$  $LoQ= \frac{10\cdot s}{b}$  Where:  *s*     is standard deviation of blank sample baseline noise in area of the chromatogram         that corresponds with the peak retention time range.  *b*      is slope of the linear regression line (dependency of peak height on specie concentration)  LoD and LoQ values in following part of this table represent the average values for all measurement days. | | | | | | | | |
| **Specie** | | | **LoD** | | | **LoQ** | | |
| As^III^ | | | (0.29 ± 0.27) µg As l^-1^ | | | (0.98 ± 0.91) µg As l^-1^ | | |
| As^V^ | | | (0.0194 ± 0.0072) µg As l^-1^ | | | (0.065 ± 0.024) µg As l^-1^ | | |
| MMA | | | (0.0200 ± 0.0071) µg As l^-1^ | | | (0.067 ± 0.024) µg As l^-1^ | | |
| DMA | | | (0.0117 ± 0.0068) µg As l^-1^ | | | (0.039 ± 0.023) µg As l^-1^ | | |
| TMAO | | | (0.0098 ± 0.0058) µg As l^-1^ | | | (0.033 ± 0.019) µg As l^-1^ | | |

***Table S14*** *Percentage of As present as the As^V^ specie - comparison between seasons*

| **Input Data** | | | | | | | | | | | | |
| --- | --- | --- | --- | --- | --- | --- | --- | --- | --- | --- | --- | --- |
|  | | | | | Data | | | | | Range | | |
| Dependent Variable | | | | | As^V^ [%] | | | | | [1*:84*] | | |
| Factor | | | | | Campaign | | | | | [1*:84*] | | |
| **Descriptive Statistics** | | | | | | | | | | | | |
|  | | N Analysis | | N Missing | | Mean | | Standard Deviation | | | SE of Mean | |
| Autumn 2022 | | 42 | | 0 | | 79.30661 | | 7.57344 | | | 1.16861 | |
| Winter 2023 | | 42 | | 0 | | 79.76887 | | 12.63913 | | | 1.95026 | |
| **One Way ANOVA** | | | | | | | | | | | | |
|  | DF | | Sum of Squares | | | | Mean Square | | F Value | | | Prob>F |
| Model | 1 | | 4.48753 | | | | 4.48753 | | 0.04134 | | | 0.83939 |
| Error | 82 | | 8901.28568 | | | | 108.55226 | |  | | |  |
| Total | 83 | | 8905.77321 | | | |  | |  | | |  |
| Null Hypothesis: The Means of all levels are equal.  Alternative Hypothesis: The means of one or more levels are different.  At the 0.05 level, the population means are not significantly different. | | | | | | | | | | | | |


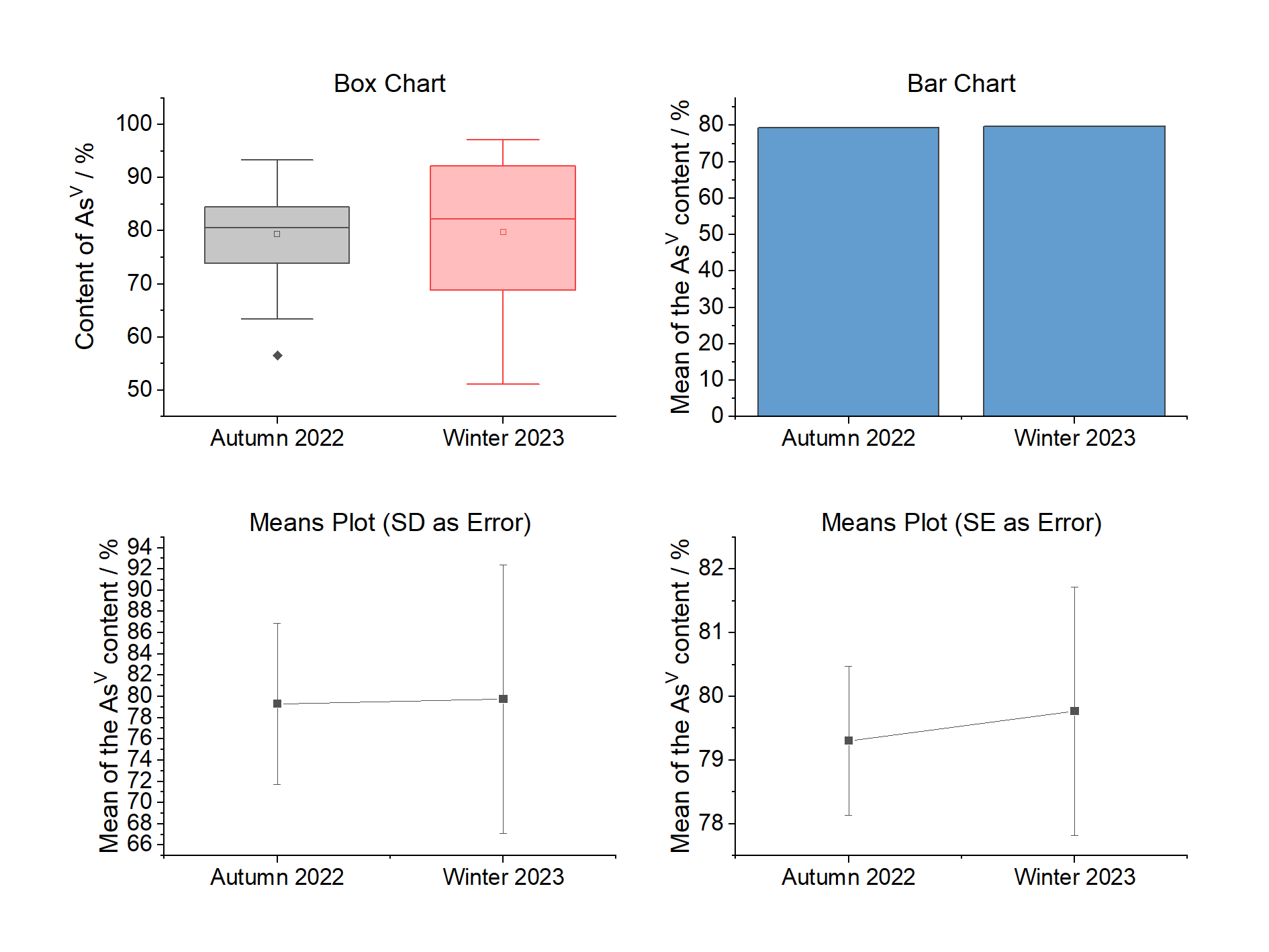


**Fig. S6** Percentage of As present as As^V^ specie. At the 0.05 level, population means do not differ significantly between seasons

**Table S15** Percentage of As present as As_org_ species - comparison between seasons

| **Input Data** | | | | | | | | | | |
| --- | --- | --- | --- | --- | --- | --- | --- | --- | --- | --- |
|  | | | Data | | | | | Range | | |
| Dependent Variable | | | As_org_ [%] | | | | | [1*:84*] | | |
| Factor | | | Campaign | | | | | [1*:84*] | | |
| **Descriptive Statistics** | | | | | | | | | | |
|  | | N Analysis | | N Missing | Mean | | Standard Deviation | | SE of Mean | |
| Autumn 2022 | | 42 | | 0 | 16.67238 | | 7.63088 | | 1.17747 | |
| Winter 2023 | | 42 | | 0 | 3.93451 | | 3.01233 | | 0.46481 | |
| **One Way ANOVA** | | | | | | | | | | |
|  | DF | | Sum of Squares | | | Mean Square | | F Value | | Prob>F |
| Model | 1 | | 3407.32022 | | | 3407.32022 | | 101.25095 | | <0.0001 |
| Error | 82 | | 2759.48281 | | | 33.65223 | |  | |  |
| Total | 83 | | 6166.80304 | | |  | |  | |  |
| Null Hypothesis: The Means of all levels are equal.  Alternative Hypothesis: The means of one or more levels are different.  At the 0.05 level, the population means are significantly different. | | | | | | | | | | |


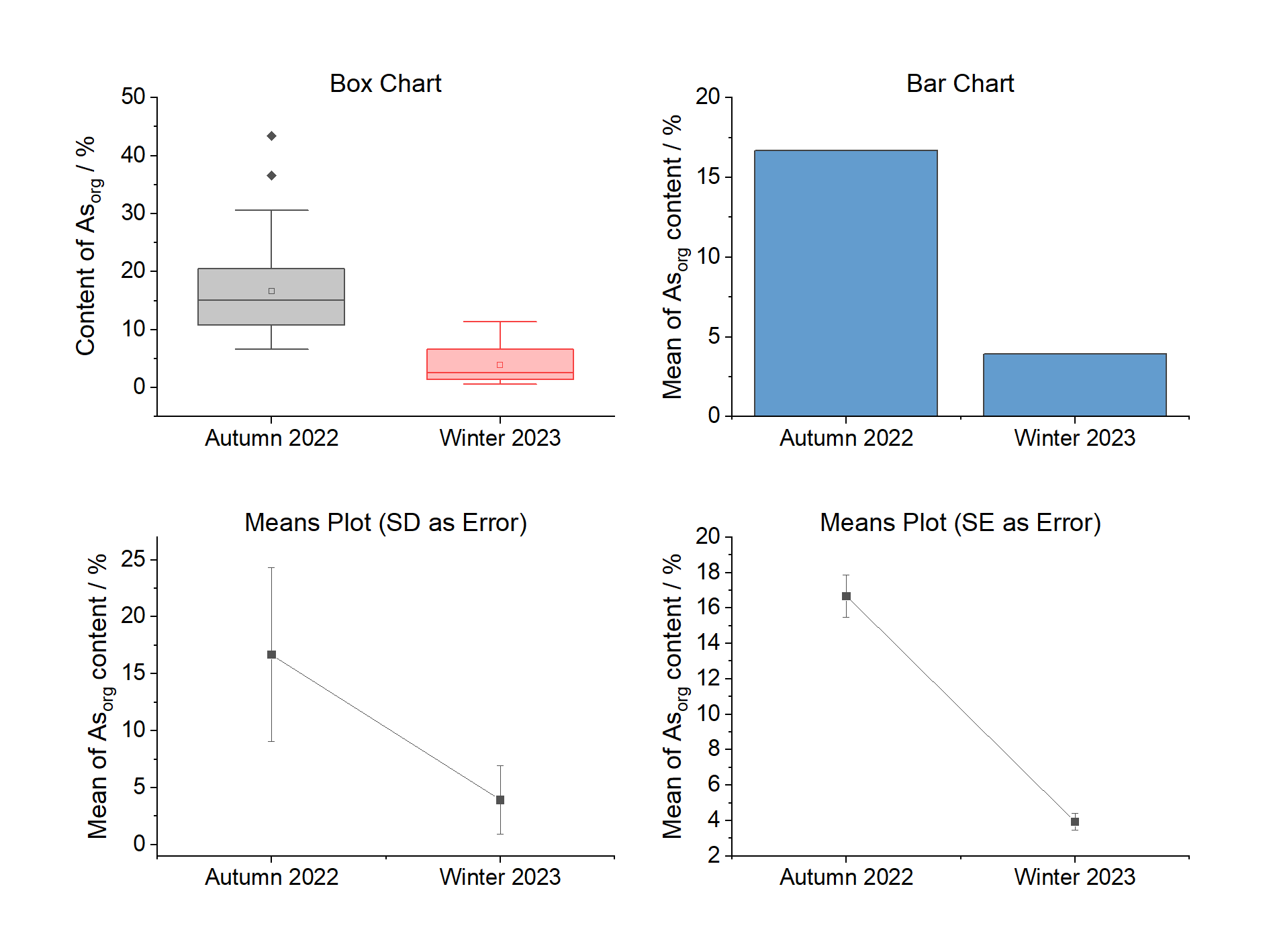


**Fig. S7** Percentage of As present as As_org_ species. At the 0.05 level, population means differ significantly between seasons

**Table S16** Percentage of As present as As_org_ specie - comparison between locations

| **Input Data** | | | | | | | | | |
| --- | --- | --- | --- | --- | --- | --- | --- | --- | --- |
|  | | | | Data | | | Range | | |
| Dependent Variable | | | | As_org_ [%] | | | [1*:84*] | | |
| Factor | | | | Location | | | [1*:84*] | | |
| **Descriptive Statistics** | | | | | | | | | |
|  | N Analysis | | N Missing | | Mean | Standard Deviation | | SE of Mean | |
| KLA | 28 | | 0 | | 7.4287 | 6.4337 | | 1.21585 | |
| KOT | 28 | | 0 | | 11.31973 | 8.39462 | | 1.58643 | |
| LUZ | 28 | | 0 | | 12.16189 | 10.1644 | | 1.92089 | |
| **One Way ANOVA** | | | | | | | | | |
|  | | DF | | Sum of Squares | | Mean Square | F Value | | Prob>F |
| Model | | 2 | | 357.02362 | | 178.51181 | 2.48881 | | 0.08934 |
| Error | | 81 | | 5809.77942 | | 71.72567 |  | |  |
| Total | | 83 | | 6166.80304 | |  |  | |  |
| Null Hypothesis: The Means of all levels are equal.  Alternative Hypothesis: The means of one or more levels are different.  At the 0.05 level, the population means are not significantly different. | | | | | | | | | |


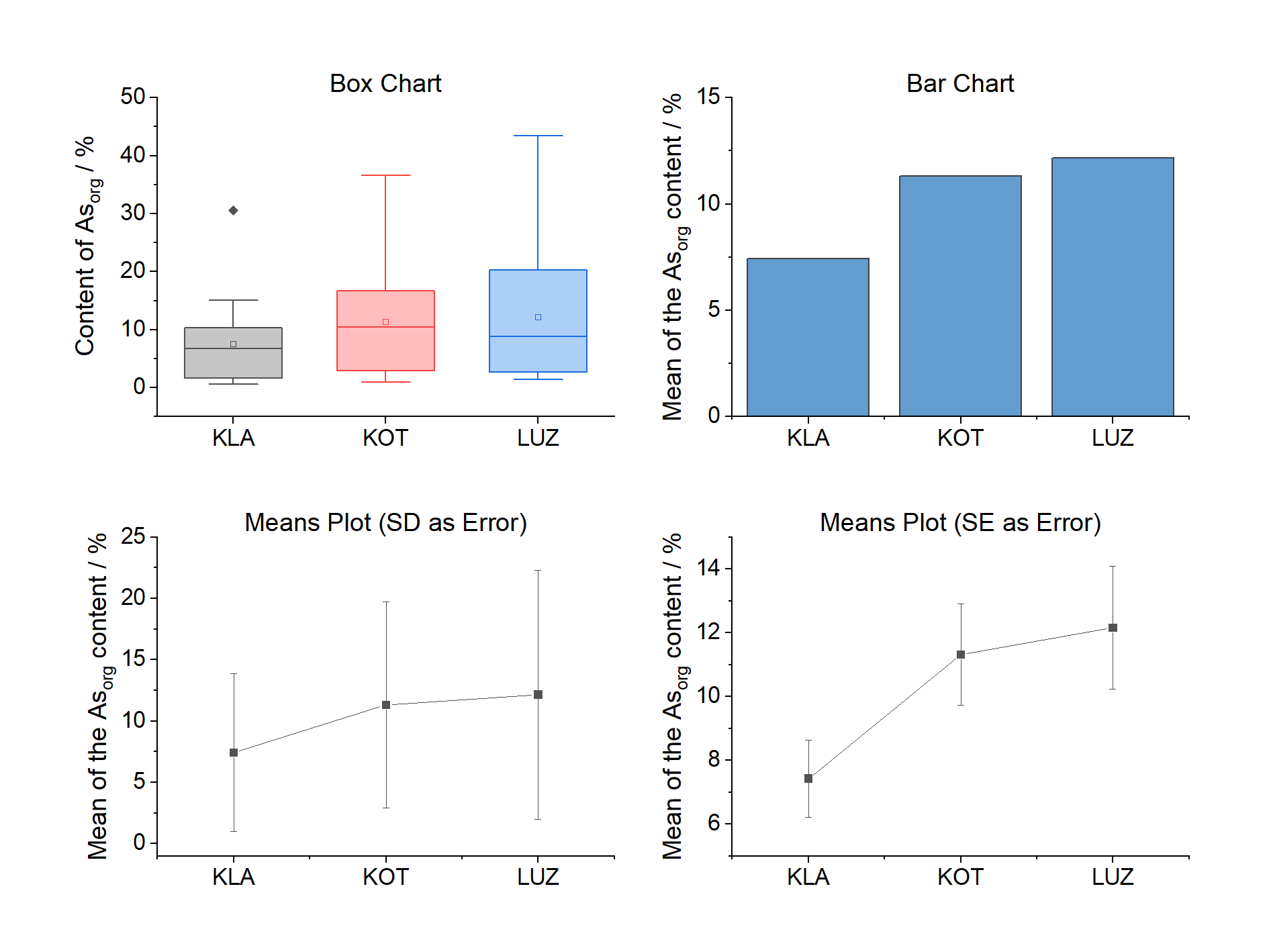


**Fig. S8** Percentage of As present as As_org_ species. At the 0.05 level, population means don’t differ significantly between locations

***Table S17*** *Percentage of As present as inorganic As species - comparison between locations*

| **Input Data** | | | | | | | | | | |
| --- | --- | --- | --- | --- | --- | --- | --- | --- | --- | --- |
|  | | | | Data | | | | Range | | |
| Dependent Variable | | | | Inorganic As [%] | | | | [1*:84*] | | |
| Factor | | | | Location | | | | [1*:84*] | | |
| **Descriptive Statistics** | | | | | | | | | | |
|  | N Analysis | | N Missing | | Mean | | Standard Deviation | | SE of Mean | |
| KLA | 28 | | 0 | | 92.5713 | | 6.4337 | | 1.21585 | |
| KOT | 28 | | 0 | | 88.68027 | | 8.39462 | | 1.58643 | |
| LUZ | 28 | | 0 | | 87.83811 | | 10.1644 | | 1.92089 | |
| **One Way ANOVA** | | | | | | | | | | |
|  | | DF | | Sum of Squares | | Mean Square | | F Value | | Prob>F |
| Model | | 2 | | 357.02362 | | 178.51181 | | 2.48881 | | 0.08934 |
| Error | | 81 | | 5809.77942 | | 71.72567 | |  | |  |
| Total | | 83 | | 6166.80304 | |  | |  | |  |
| Null Hypothesis: The Means of all levels are equal.  Alternative Hypothesis: The means of one or more levels are different.  At the 0.05 level, the population means are not significantly different. | | | | | | | | | | |


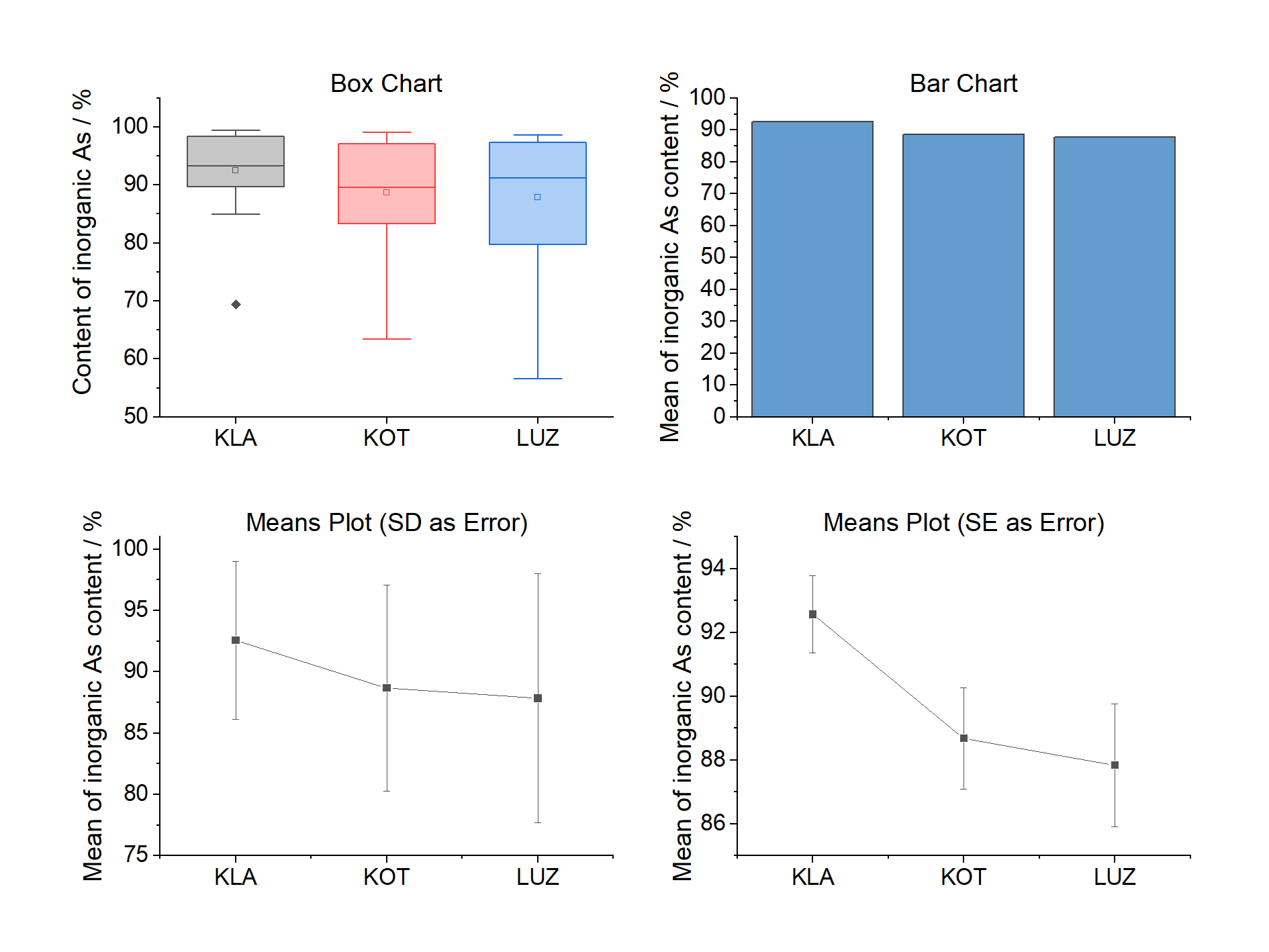


**Fig. S9** Percentage of As present as inorganic As species. At the 0.05 level, population means don’t differ significantly between locations

**Table S18** Percentage of As present as As^III^ specie - comparison between locations

| **Input Data** | | | | | | | | | | |
| --- | --- | --- | --- | --- | --- | --- | --- | --- | --- | --- |
|  | | | | Data | | | | Range | | |
| Dependent Variable | | | | As^III^ [%] | | | | [1*:84*] | | |
| Factor | | | | Location | | | | [1*:84*] | | |
| **Descriptive Statistics** | | | | | | | | | | |
|  | N Analysis | | N Missing | | Mean | | Standard Deviation | | SE of Mean | |
| KLA | 28 | | 0 | | 8.06234 | | 9.17008 | | 1.73298 | |
| KOT | 28 | | 0 | | 13.58059 | | 14.90507 | | 2.81679 | |
| LUZ | 28 | | 0 | | 8.83353 | | 9.55054 | | 1.80488 | |
| **One Way ANOVA** | | | | | | | | | | |
|  | | DF | | Sum of Squares | | Mean Square | | F Value | | Prob>F |
| Model | | 2 | | 500.08253 | | 250.04127 | | 1.88727 | | 0.15809 |
| Error | | 81 | | 10731.5346 | | 132.48808 | |  | |  |
| Total | | 83 | | 11231.61714 | |  | |  | |  |
| Null Hypothesis: The Means of all levels are equal.  Alternative Hypothesis: The means of one or more levels are different.  At the 0.05 level, the population means are not significantly different. | | | | | | | | | | |


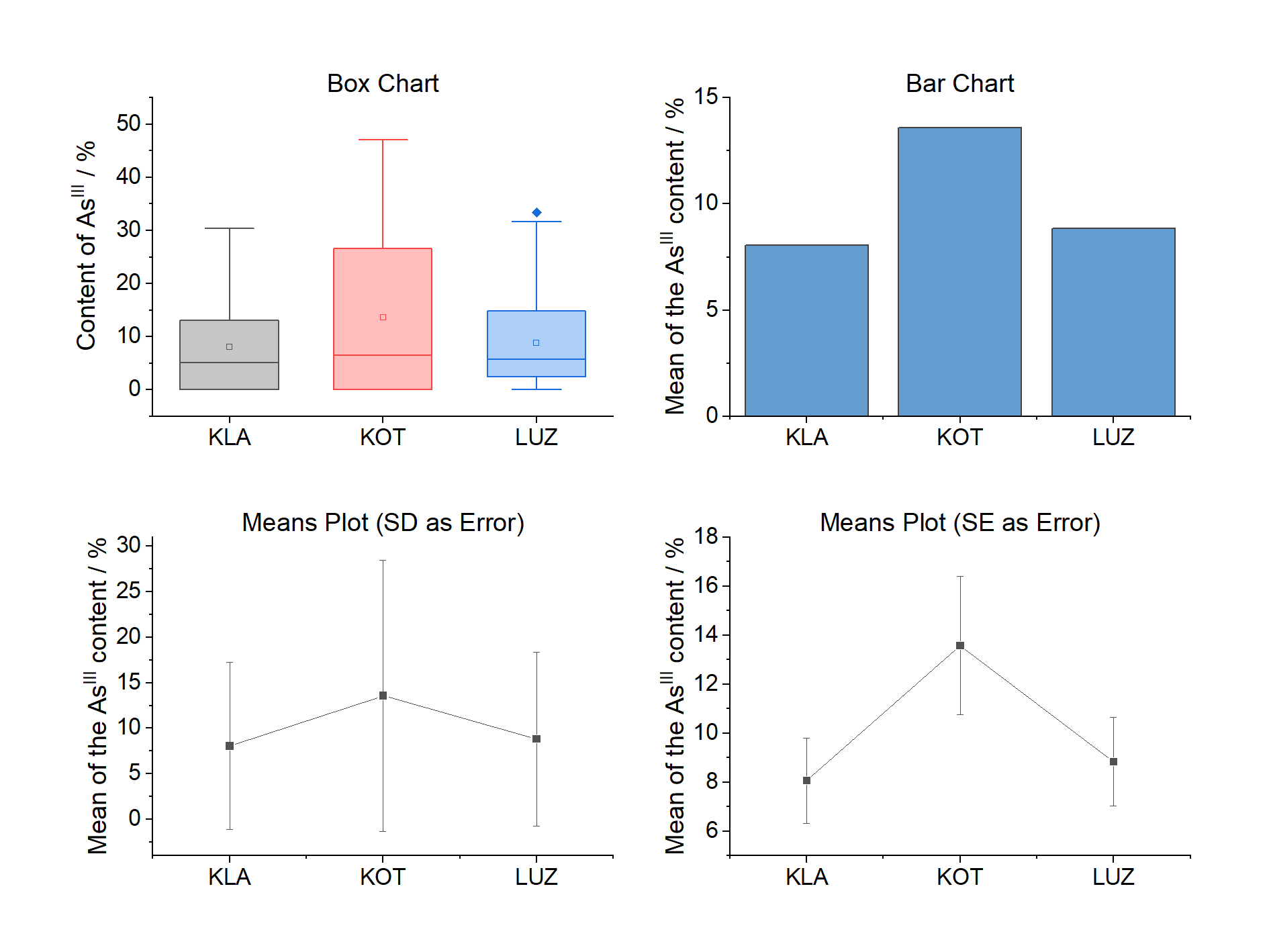


**Fig. S10** Percentage of As present as As^III^ specie. At the 0.05 level, population means don’t differ significantly between locations

**Table S19** Percentage of As present as As^V^ specie - comparison between locations

| **Input Data** | | | | | | | | | | |
| --- | --- | --- | --- | --- | --- | --- | --- | --- | --- | --- |
|  | | | | Data | | | | Range | | |
| Dependent Variable | | | | As^V^ [%] | | | | [1*:84*] | | |
| Factor | | | | Location | | | | [1*:84*] | | |
| **Descriptive Statistics** | | | | | | | | | | |
|  | N Analysis | | N Missing | | Mean | | Standard Deviation | | SE of Mean | |
| KLA | 28 | | 0 | | 84.50896 | | 7.42568 | | 1.40332 | |
| KOT | 28 | | 0 | | 75.09968 | | 11.37558 | | 2.14978 | |
| LUZ | 28 | | 0 | | 79.00458 | | 9.94736 | | 1.87987 | |
| **One Way ANOVA** | | | | | | | | | | |
|  | | DF | | Sum of Squares | | Mean Square | | F Value | | Prob>F |
| Model | | 2 | | 1251.42206 | | 625.71103 | | 6.62141 | | 0.00217 |
| Error | | 81 | | 7654.35115 | | 94.49816 | |  | |  |
| Total | | 83 | | 8905.77321 | |  | |  | |  |
| Null Hypothesis: The Means of all levels are equal.  Alternative Hypothesis: The means of one or more levels are different.  At the 0.05 level, the population means are significantly different. | | | | | | | | | | |


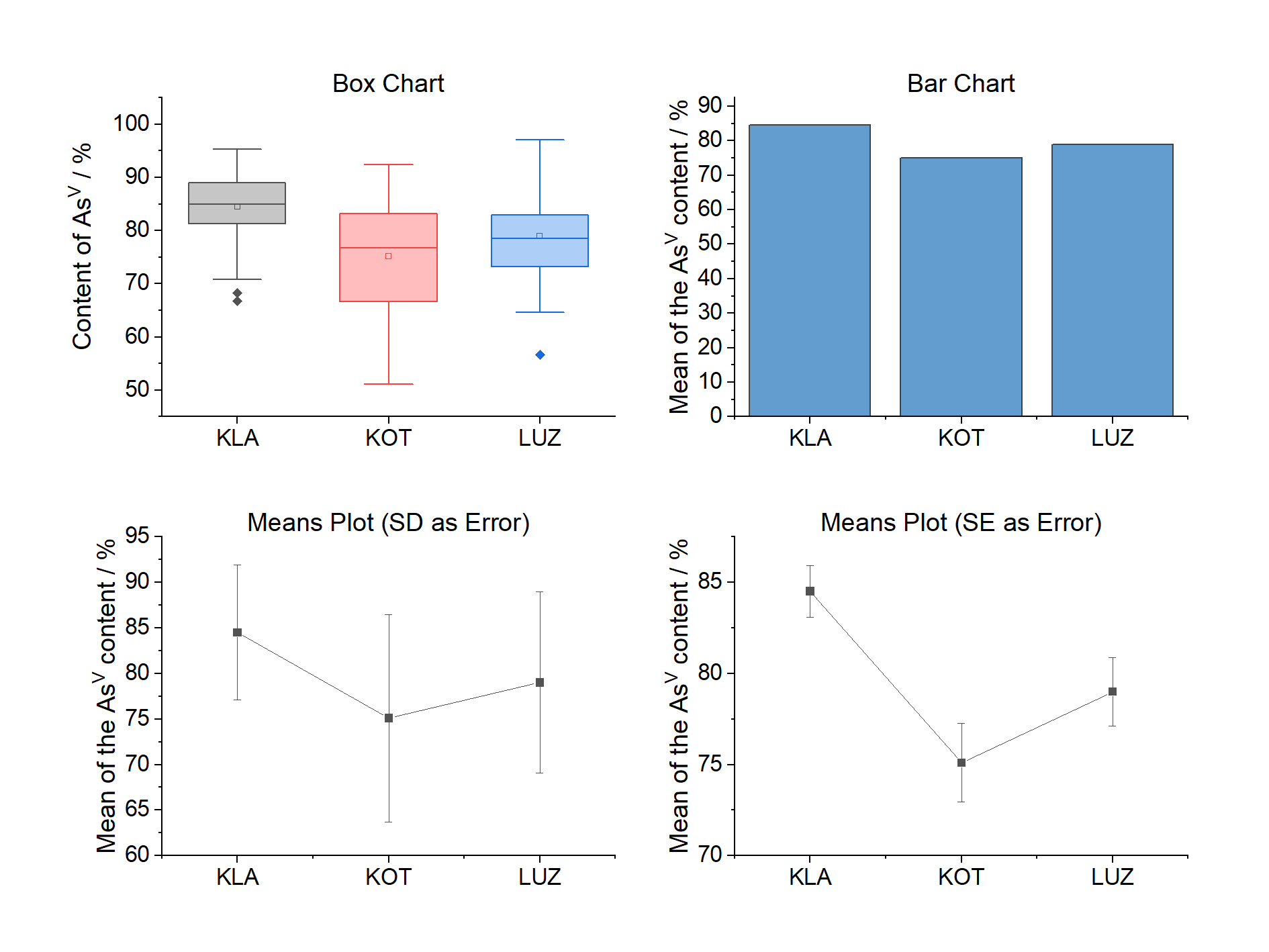


**Fig. S11** Percentage of As present as As^V^ specie. At the 0.05 level, population means differ significantly between locations

## Arsenic water extraction efficiency

The water extraction efficiency of As from the 83 successfully analysed PM_10_ samples was 84.1 ± 16.7%. This result is based on the difference between the As_Total_ content analysed in the mineralised sample and in the water extract of the same sample (Table S10 and Fig. S3), assuming that all of As is transferred to the liquid phase by the mineralisation process.


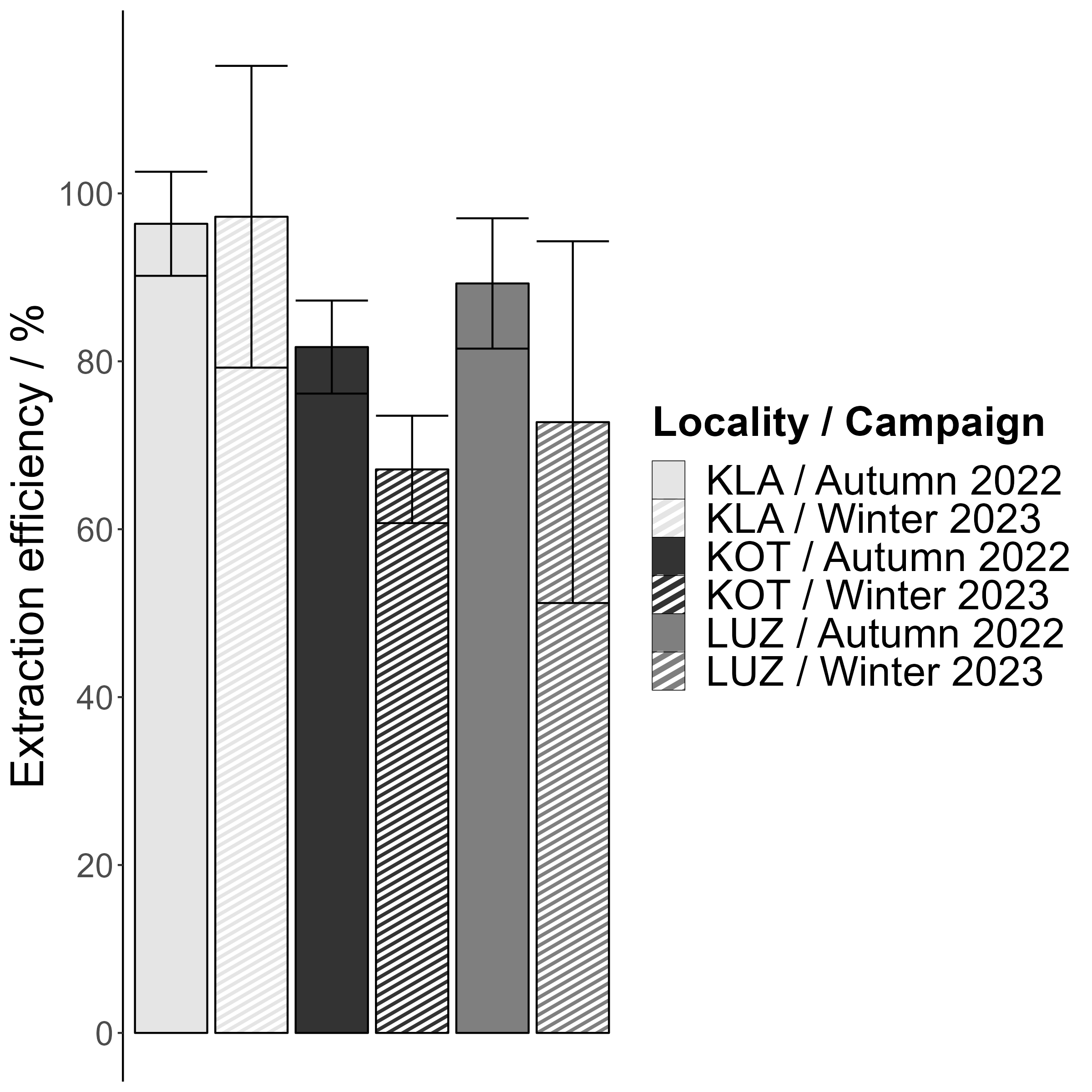


**Fig. S12** Comparison of extraction efficiency for different seasons and sampling locations

The water extraction efficiency was found to be location-dependent, indicating a change in the composition of PM due to its different sources. While samples from the background location KLA were extracted with an efficiency of 96.8 ± 13.2%, the samples from the city intersection KOT were extracted only with an efficiency of 74.4 ± 9.5%. The park location LUZ was in the middle with an extraction efficiency of 81.0 ± 18.0% (Fig. S12). Tanda et al. (2019, 2020) discussed that this trend may be related to the particle aerodynamic diameter. They also observed a lower extraction efficiency during the winter season, when the particles increased in size. Our results, with 89.1 ± 8.8% of extraction efficiency in autumn and 79.0 ± 20.9% in winter, show an analogous behaviour.

**Table S20** As_Total_ concentrations in water-extracted PM_10_ samples collected during both sampling campaigns in units of units pg As per m^3^ of filtered air

| Day | Locality | | | | | |
| --- | --- | --- | --- | --- | --- | --- |
|  | KLA | | LUZ | | KOT | |
|  | Campaign | | Campaign | | Campaign | |
|  | Autumn 2022 | Winter 2023 | Autumn 2022 | Winter 2023 | Autumn 2022 | Winter 2023 |
| 1 | 543 ± 10 | 413 ± 14 | 876.7 ± 4.8 | 278.4 ± 4.2 | 694 ± 14 | 258.1 ± 5.0 |
| 2 | 608 ± 12 | 228.0 ± 8.0 | 879.9 ± 7.6 | 197.7 ± 2.6 | 773 ± 19 | 169.7 ± 4.6 |
| 3 | 412.4 ± 6.0 | 230.2 ± 7.1 | 567.6 ± 5.7 | 198.9 ± 2.4 | 430.0 ± 5.9 | 180.2 ± 5.0 |
| 4 | 328.8 ± 6.9 | 664 ± 11 | 338.6 ± 5.5 | 1078 ± 15 | 288 ± 10 | 928 ± 99 |
| 5 | 503.8 ± 4.4 | 255.3 ± 5.6 | 575 ±11 | 229.2 ± 1.5 | 532 ± 13 | 235.1 ± 4.0 |
| 6 | 614 ± 16 | 717 ± 14 | 837.2 ± 4.9 | 522.6 ± 6.7 | 673 ± 12 | 455.3 ± 6.8 |
| 7 | 559 ± 14 | 1310.5 ± 8.5 | 647 ± 14 | 1839 ± 43 | 552 ± 17 | 1234 ± 15 |
| 8 | 1042 ± 20 | 1891 ± 26 | 1246 ± 21 | 2616 ± 31 | 1124 ± 20 | 1810 ± 14 |
| 9 | 842 ± 16 | 2025 ± 28 | 958 ± 17 | 2422 ± 38 | 896 ± 93 | 1727 ± 15 |
| 10 | 1022 ± 22 | 1055 ± 15 | 984 ± 14 | * | 928 ± 13 | 1202 ± 13 |
| 11 | 1084 ± 13 | 813 ± 16 | 1036 ± 19 | 1515 ± 27 | 929 ± 16 | 939 ± 16 |
| 12 | 923 ± 11 | 1460 ± 18 | 1046.1 ± 6.1 | 2326 ± 16 | 904 ± 23 | 1369 ± 25 |
| 13 | 943 ± 12 | 977.0 ± 9.2 | 811.1 ± 5.6 | 1057 ± 17 | 768 ± 17 | 871 ± 17 |
| 14 | 757 ± 13 | 1057 ± 16 | 680 ± 16 | 1434 ± 22 | 580.4 ± 8.0 | 927 ± 19 |
| * Unable to analyse | | | | | | |


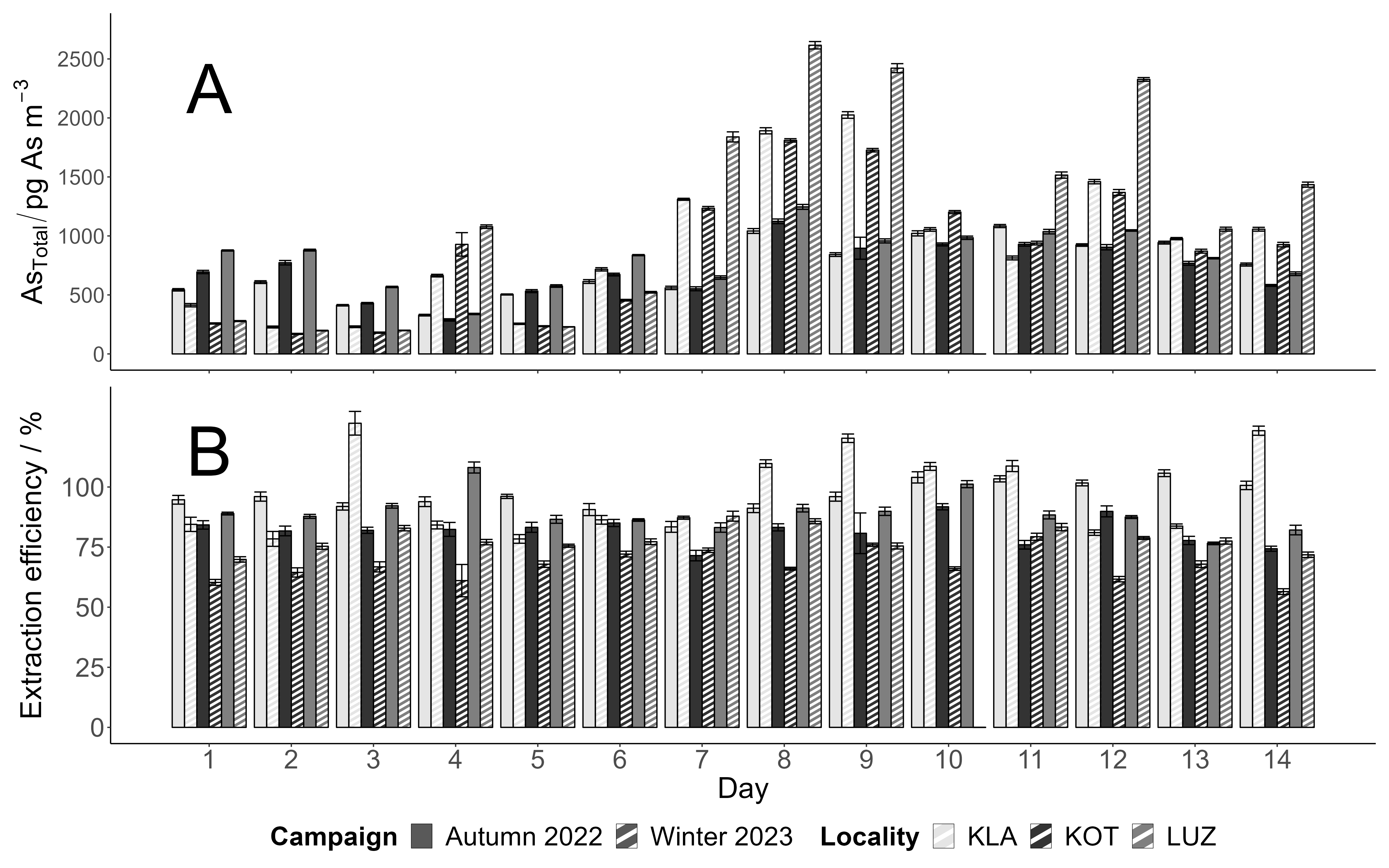


**Fig. S13** Total As concentrations in water-extracted samples collected during both sampling campaigns for the three chosen localities in units of pg As per m^3^ of filtered air (A) and extraction efficiencies for every sample (B). Source data are in Tables S7 and S20. The error bars represent standard deviations for each measurement.

## Supplementary Information References

Cheng J, Karambelkar B, Xie Y, 2022. leaflet: Create Interactive Web Maps with the JavaScript “Leaflet” Library. R package version 2.1.1.

CHMI, 2022 [WWW Document], URL https://www.chmi.cz/files/portal/docs/uoco/web_generator/plants/brno_mesto_CZ.html (accessed 11.13.23). (in Czech)

OpenStreetMap [WWW Document], n.d. URL https://www.openstreetmap.org/copyright (accessed 7.13.23).

Tanda, S., Gingl, K., Ličbinský, R., Hegrová, J., Goessler, W., 2020. Occurrence, Seasonal Variation, and Size Resolved Distribution of Arsenic Species in Atmospheric Particulate Matter in an Urban Area in Southeastern Austria. Environ Sci Technol 54, 5532–5539. https://doi.org/10.1021/acs.est.9b07707

Tanda, S., Ličbinský, R., Hegrová, J., Faimon, J., Goessler, W., 2019. Arsenic speciation in aerosols of a respiratory therapeutic cave: A first approach to study arsenicals in ultrafine particles. Science of The Total Environment 651, 1839–1848. https://doi.org/10.1016/J.SCITOTENV.2018.10.102

WHO global air quality guidelines. Particulate matter (PM_2.5_ and PM_10_), ozone, nitrogen dioxide, sulfur dioxide and carbon monoxide. Geneva: World Health Organization; 2021. Licence: CC BY-NC-SA 3.0 IGO., 2021.
